# On optimal Bayesian classification and risk estimation under multiple classes

**DOI:** 10.1186/s13637-015-0028-3

**Published:** 2015-10-24

**Authors:** Lori A. Dalton, Mohammadmahdi R. Yousefi

**Affiliations:** 1 Department of Electrical and Computer Engineering, The Ohio State University, Columbus, 43210 OH USA; 2 Department of Biomedical Informatics, The Ohio State University, Columbus, 43210 OH USA

**Keywords:** Risk estimation, Multi-class classification, Bayesian estimation, Genomics, Minimum mean-square error, Small samples

## Abstract

A recently proposed optimal Bayesian classification paradigm addresses optimal error rate analysis for small-sample discrimination, including optimal classifiers, optimal error estimators, and error estimation analysis tools with respect to the probability of misclassification under binary classes. Here, we address multi-class problems and optimal expected risk with respect to a given risk function, which are common settings in bioinformatics. We present Bayesian risk estimators (BRE) under arbitrary classifiers, the mean-square error (MSE) of arbitrary risk estimators under arbitrary classifiers, and optimal Bayesian risk classifiers (OBRC). We provide analytic expressions for these tools under several discrete and Gaussian models and present a new methodology to approximate the BRE and MSE when analytic expressions are not available. Of particular note, we present analytic forms for the MSE under Gaussian models with homoscedastic covariances, which are new even in binary classification.

## Introduction

Classification in biomedicine is often constrained by small samples so that understanding properties of the error rate is critical to ensure the scientific validity of a designed classifier. While classifier performance is typically evaluated by employing distribution-free training-data error estimators such as cross-validation, leave-one-out, or bootstrap, a number of studies have demonstrated that these methods are highly problematic in small-sample settings [1]. Under real data and even under simple synthetic Gaussian models, cross-validation has been shown to suffer from a large variance [2] and often has nearly zero correlation, or even negative correlation, with the true error [3, 4]. Among other problems, this directly leads to severely optimistic reporting biases when selecting the best results among several datasets [5] or when selecting the best classification rule among several candidates [6] and difficulties with performance reproducibility [7].

Furthermore, there are typically no accuracy guarantees for error estimators when applied under small samples. *Distribution-free* bounds on the *mean-square error* (MSE) or its square root, the *root-mean-square* (RMS), of an error estimator with respect to the true error rate are typically either unavailable or unhelpful under small samples. Consider leave-one-out error estimation for a discrete histogram rule that breaks ties with class 0. The following is a distribution-free RMS bound [8]:

(1) $$ \text{RMS}(\widehat{\varepsilon }_{\text{loo}}(\mathcal{S}) \,| \, \theta) \leq \sqrt{\frac{1+6/e}{n}+\frac{6}{\sqrt{\pi \left(n-1\right) }}},   $$

where $\mathcal {S}$ is a random sample, *θ* is a feature-label distribution, and *n* is the sample size. To guarantee an RMS less than 0.5 for all distributions, this bound indicates that a sample size of at least *n*=209 would be required. Typically, the error of a classifier should be between 0 and 0.5 so that an RMS of 0.5 is trivially guaranteed.

Rather than a distribution-free approach, recent work takes a Bayesian approach to address these problems. The idea is to assume the true distributions characterizing classes in the population are members of an uncertainty class of models. We also assume that members of the uncertainty class are weighted by a *prior* distribution, and after observing a sample, we update the prior to a *posterior* distribution. For a given classifier we may find an optimal MSE error estimator, called a *Bayesian error estimator* (BEE) [9, 10] and evaluate the MSE of any arbitrary error estimator [11, 12]. These quantities are found by conditioning on the sample in hand and averaging with respect to the unknown population distribution via the posterior, rather than by conditioning on the distribution and averaging over random samples as in (1). Not only does the Bayesian framework supply more powerful error estimators, but the sample-conditioned MSE allows us to evaluate the accuracy of error estimation. The Bayesian framework also facilitates *optimal Bayesian classification* (OBC), which provides decision boundaries to minimize the BEE [13, 14]. In this way, the Bayesian framework can be used to optimize both error estimation and classification.

Classifier design and analysis in the Bayesian framework have previously been developed for binary classification with respect to the probability of misclassification. However, it is common in small-sample classification problems to be faced with classification under multiple classes and for different types of error to be associated with different levels of risk or loss. A few classical classification algorithms naturally permit multiple classes and arbitrary loss functions; for example, a *plug-in rule* takes the functional form for an optimal Bayes decision rule under a given modeling assumption and substitutes sample estimates of model parameters in place of the true parameters. This can be done with linear discriminant analysis (LDA) and quadratic discriminant analysis (QDA) for multiple classes with arbitrary loss functions, which essentially assume that the underlying class-conditional densities are Gaussian with equal or unequal covariances, respectively. Most training-data error estimation methods, for instance, cross-validation, can also be generalized to handle multiple classes and arbitrary loss functions. However, it is expected that the same difficulties encountered under binary classes with simple zero-one loss functions (where the expected risk reduces to the probability of misclassification) will carry over to the more general setting, as they have in ROC curve estimation [15].

Support vector machines (SVM) are inherently binary but can be adapted to incorporate penalties that influence risk by implementing slack terms or applying a shrinkage or robustifying objective function [16, 17]. It is also common to construct multi-class classifiers from binary classifiers using the popular “one-versus-all” or “all-versus-all” strategies [18]. The former method builds several binary classifiers by discriminating one class, in turn, against all others, and at a given test point reports the class corresponding to the highest classification score. The latter discriminates between each combination of pairs of classes and reports a majority vote. However, it is unclear how one may assess the precise effect of these adaptations on the expected risk.

We are thus motivated to generalize the BEE, sample-conditioned MSE, and OBC to treat multiple classes with arbitrary loss functions. We will present analogous concepts of *Bayesian risk estimation* (BRE), the sample-conditioned MSE for risk estimators, and *optimal Bayesian risk classification* (OBRC). We will show that the BRE and OBRC can be represented in the same form as the expected risk and Bayes decision rule with unknown true densities replaced by *effective densities*. This approach is distinct from the simple plug-in rule discussed earlier, since the form of the effective densities may not be the same as the individual densities represented in the uncertainty class. We will also develop an interpretation of the conditional MSE based on an *effective joint density*, which is new even under binary classes with a zero-one loss function.

Furthermore, we will provide analytic solutions under several models: discrete spaces with Dirichlet priors (discrete models) and Gaussian distributions with known, independent scaled identity, independent arbitrary, homoscedastic scaled identity, and homoscedastic arbitrary covariance models, all with conjugate priors (Gaussian models). We provide expressions for the BRE and conditional MSE for arbitrary classification in the discrete model and binary linear classification in the Gaussian model. The analytic form that we provide for the MSE of arbitrary error estimators under homoscedastic models is completely new without an analog in prior work under binary classification and zero-one loss. For models in which an analytic form for the BRE and conditional MSE are unavailable, for instance, under multi-class or non-linear classification in the Gaussian model, we also discuss efficient methods to approximate these quantities. In particular, we present a new computationally efficient method to approximate the conditional MSE based on the effective joint density.

## Notation

We denote random quantities with capital letters, e.g., *Y*; realizations of random variables with lowercase letters, e.g., *y*; and vectors in bold, e.g., **X** and **x**. Matrices will generally be in bold upper case, e.g., **S**. Spaces will be denoted by a stylized font, e.g., $\mathcal {X}$. Distributions with conditioning will be made clear through the function arguments; for instance, we write the distribution of **X** given *Y* as *f*(**x** | *y*). The probability space of expectations will be made clear by denoting random quantities in the expectation and conditioning, e.g., the expectation of *Y* conditioned on the random variable **X** and the event **C**=**c** is denoted by E[*Y* | **X**,**c**]. When the region of integration in an integral is omitted then this region is the whole space. Any exceptions in notation will be defined throughout.

## Bayes decision theory

We next review concepts from classical Bayes decision theory. Consider a classification problem in which we are to predict one of *M* classes, *y*=0,…,*M*−1, from a sample drawn in feature space $\mathcal {X}$. Let **X** and *Y* denote a random feature vector and its corresponding random label. Let *f*(*y* | **c**) be the probability mass function of *Y*, parameterized by a vector **c**, and for each *y*, let *f*(**x** | *y*,*θ*_*y*_) be the *class-y-conditional density* of **X**, parameterized by a vector *θ*_*y*_. The full feature-label distribution is parameterized by **c** and *θ*={*θ*_0_,…,*θ*_*M*−1_}.

Let *λ*(*i*,*y*) be a loss function quantifying a penalty in predicting label *i* when the true label is *y*. The *conditional risk* in predicting label *i* for a given point, **x**, is defined as

(2) $$\begin{array}{*{20}l} R(i, \mathbf{x}, \mathbf{c}, \theta) &= \text{E}[\lambda(i, Y) \, | \, \mathbf{x}, \mathbf{c}, \theta]  \\ &= \sum_{y = 0}^{M-1} \lambda(i, y) f(y \, | \,\mathbf{x}, \mathbf{c}, \theta)  \\ &= \frac{\sum_{y = 0}^{M-1} \lambda(i, y) f(y \, | \, \mathbf{c}) f(\mathbf{x} \, | \, y, \theta_{y})} {\sum_{y = 0}^{M-1} f(y \, | \, \mathbf{c}) f(\mathbf{x} \, | \, y, \theta_{y})}.  \end{array} $$

The *expected risk* of a given classification rule, $\psi :\mathcal {X} \rightarrow \{0, \ldots, M-1\}$, is given by

(3) $$\begin{array}{*{20}l} R(\psi, \mathbf{c}, \theta) &= \text{E}[R(\psi(\mathbf{X}), \mathbf{X}, \mathbf{c}, \theta) \, | \, \mathbf{c}, \theta]  \\ &= \sum_{y = 0}^{M-1} \sum_{i = 0}^{M-1} \lambda(i, y) f(y \, | \, \mathbf{c}) \varepsilon^{i, y}(\psi, \theta_{y}),  \end{array} $$

where

(4) $$ \varepsilon^{i, y}(\psi, \theta_{y}) = \int_{\Gamma_{i}} f(\mathbf{x} \, | \, y, \theta_{y}) d\mathbf{x} = \text{P}(\mathbf{X} \in \Gamma_{i} \, | \, y, \theta_{y})   $$

is the probability that a class-*y* point will be assigned class *i* by the classifier *ψ*, and the $\Gamma _{i} = \{\mathbf {x} \in \mathcal {X}: \psi (\mathbf {x}) = i\}$ partition the sample space into decision regions.

A *Bayes decision rule* (BDR) minimizes expected risk or, equivalently, the conditional risk at each fixed point **x**:

(5) $$\begin{array}{*{20}l} \psi_{\text{BDR}}\left(\mathbf{x}\right) &= \text{arg} \min_{i \in \{0, \ldots, M-1\}} R(i, \mathbf{x}, \mathbf{c}, \theta).  \end{array} $$

By convention, we break ties with the lowest index, *i*∈{0,…,*M*−1}, minimizing *R*(*i*,**x**,**c**,*θ*).

## Optimal Bayesian risk classification

In practice, the feature-label distribution is unknown so that we must train a classifier and estimate risk or error with data. The Bayesian framework resolves this by assuming the true feature-label distribution is a member of a parameterized uncertainty class. In particular, assume that **c** is the probability mass function of *Y*, that is, **c**={*c*_0_,…,*c*_*M*−1_}∈*Δ* ^*M*−1^, where *f*(*y* | **c**)=*c*_*y*_ and *Δ* ^*M*−1^ is the standard *M*−1 simplex defined by *c*_*y*_∈[0,1] for *y*∈{0,…,*M*−1} and $\sum _{y = 0}^{M-1} c_{y} = 1$. Also assume $\theta _{y} \in \mathcal {T}_{y}$ for some parameter space $\mathcal {T}_{y}$, and $\theta \in \mathcal {T} = \mathcal {T}_{0} \times \ldots \times \mathcal {T}_{M-1}$. Let **C** and *Θ* denote random vectors for parameters **c** and *θ*, respectively. Finally, assume **C** and *Θ* are independent prior to observing data and assign *prior* probabilities, *π*(**c**) and *π*(*θ*).

Priors quantify uncertainty we have about the distribution before observing the data. Although non-informative priors may be used as long as the posterior is normalizable, informative priors can supplement the classification problem with information to improve performance when the sample size is small. This is key for problems with limited or expensive data. Under mild regularity conditions, as we observe sample points, this uncertainty converges to a certainty on the true distribution parameters, where more informative priors may lead to faster convergence [12]. For small samples, the performance of Bayesian methods depends heavily on the choice of prior. Performance tends to be modest but more robust with a non-informative or weakly informative prior. Conversely, informative priors offer the potential for great performance improvement, but if the true population distribution is not well represented in the prior, then performance may be poor. This trade-off is acceptable as long as the prior is an accurate reflection of available scientific knowledge so that one is reasonably sure that catastrophic results will not occur. If multiple models are scientifically reasonable but result in different inferences, and if it is not possible to determine which model is best from data or prior knowledge, then the range of inferences must be considered [19]. For the sake of illustration, in simulations, we will utilize either low-information priors or a simple prior construction method for microarray data, although modeling and prior construction remain important problems [20].

Let *S* be a sample, that is, a realization of *n* independent labeled points drawn from $\mathcal {X}$. Also let $\mathbf {x}_{i}^{y}$ denote the *i*th sample point in class *y* and *n*_*y*_ denote the number of sample points observed from class *y*. Given a sample, the priors are updated to *posterior* densities:

(6) $$\begin{array}{*{20}l} f(\mathbf{c}, \theta \,| \, S) &\propto \pi(\mathbf{c}) \pi(\theta) \prod_{y = 0}^{M-1} \prod_{i = 1}^{n_{y}} f(\mathbf{x}_{i}^{y}, y \,| \, \mathbf{c}, \theta_{y}), \end{array} $$

where the product on the right is the usual *likelihood function*. Since $f(\mathbf {x}_{i}^{y}, y \,| \, \mathbf {c}, \theta _{y}) = c_{y} \,f(\mathbf {x}_{i}^{y} \, | \, y, \theta _{y})$, we may write *f*(**c**,*θ* | *S*)=*f*(**c** | *S*) *f*(*θ* | *S*), where

(7) $$ f(\mathbf{c} \, | \, S) \propto \pi(\mathbf{c}) \prod_{y = 0}^{M-1} (c_{y})^{n_{y}}   $$

and

(8) $$ f(\theta \, | \,S) \propto \pi(\theta) \prod_{y = 0}^{M-1} \prod_{i = 1}^{n_{y}} f(\mathbf{x}_{i}^{y} \, | \, y, \theta_{y})   $$

are marginal posteriors of **C** and *Θ*. Thus, independence between **C** and *Θ* is preserved in the posterior. Constants of proportionality are found by normalizing the integral of posteriors to 1. When the prior density is proper, this all follows from Bayes’ rule; otherwise, (7) and (8) are taken as definitions, where we require posteriors to be proper.

*f*(**c** | *S*) depends on the prior and sampling method used. For instance, if **C** is known, then *π*(**c**) and *f*(**c** | *S*) are both point masses at the known value of **C**. Under separate sampling, in which the number of sample points in each class is fixed to an arbitrary value prior to sampling, *f*(**c** | *S*)=*π*(**c**). Under random sampling, the sample size is fixed at *n* and the number of points observed from each class is determined by independent draws from the feature-label distribution. Given a Dirichlet prior on **C** with hyperparameters **α**={*α*_0_,…,*α*_*M*−1_}, a special case being *α*_0_=…=*α*_*M*−1_=1 for a uniform distribution on *Δ* ^*M*−1^, then under random sampling the posterior on **C** is still Dirichlet with hyperparameters $\alpha _{y}^{\ast }=\alpha _{y}+n_{y}$. Defining $\alpha _{+}^{\ast } = \sum _{i = 0}^{M-1} \alpha _{i}^{\ast }$, we also have for *y*≠*z*,

(9) $$\begin{array}{*{20}l} \text{E}\left[ C_{y} \, | \, S\right] &= \frac{\alpha_{y}^{\ast }}{\alpha_{+}^{\ast}},  \end{array} $$

(10) $$\begin{array}{*{20}l} \text{E}\left[ {C_{y}^{2}} \, | \, S \right] &= \frac{\alpha_{y}^{\ast} \left(1 + \alpha_{y}^{\ast} \right)} {\alpha_{+}^{\ast}\left(1 + \alpha_{+}^{\ast}\right)},  \end{array} $$

(11) $$\begin{array}{*{20}l} \text{E}\left[ C_{y} C_{z} \, |\, S \right] &= \frac{\alpha_{y}^{\ast} \alpha_{z}^{\ast}} {\alpha_{+}^{\ast}\left(1 + \alpha_{+}^{\ast}\right)}.  \end{array} $$

### Bayesian risk estimation

We define the BRE to be the minimum mean-square error (MMSE) estimate of the expected risk or, equivalently, the conditional expectation of the expected risk given observations. Given a sample, *S*, and a classifier, *ψ*, that is not informed by *θ*, thanks to posterior independence between **C** and *Θ*, the BRE is given by,

(12) $$\begin{array}{*{20}l} \widehat{R}(\psi, S) &= \text{E}[R(\psi, \mathbf{C}, \Theta) \, | \,S]  \\ &= \sum_{y = 0}^{M-1} \sum_{i = 0}^{M-1} \lambda(i, y) \text{E}[f(y \, | \, \mathbf{C}) \, | \, S] \text{E} [\varepsilon^{i, y}\left(\psi, \Theta \right) \, | \, S].  \end{array} $$

If we assume that {**X**,*Y*} and $\mathcal {S}$ are independent given **C** and *Θ*, then

(13) $$\begin{array}{*{20}l} f(y \, | \, S) &= \int f(y \, | \, \mathbf{c}) f(\mathbf{c} \, | \, S) d\mathbf{c}  \\ &= \text{E} \left[ f(y \,| \, \mathbf{C}) \, | \, S \right], \end{array} $$

(14) $$\begin{array}{*{20}l} f\left(\mathbf{x} \, | \, y, S\right) &=\int f\left(\mathbf{x} \, | \, y, \theta_{y}\right) f\left(\theta_{y} \, | \, S\right) d\theta_{y}  \\ &= \text{E}[f(\mathbf{x} \, | \, y, \Theta_{y}) \, | \, S ].  \end{array} $$

We may thus write the BRE in (12) as

(15) $$\begin{array}{*{20}l} \widehat{R}(\psi, S) &= \sum_{y = 0}^{M-1} \sum_{i = 0}^{M-1} \lambda(i, y) f(y \,| \, S) \widehat{\varepsilon }^{i, y}(\psi, S),  \end{array} $$

where $\widehat {\varepsilon }^{i, y}(\psi, S) = \text {E} [\varepsilon ^{i, y}\left (\psi, \Theta \right) \, | \, S]$ is the posterior probability of assigning a class-*y* point to class *i*,

(16) $$\begin{array}{*{20}l} \widehat{\varepsilon }^{i, y}(\psi, S) &= \text{E}\left[ \int_{\Gamma_{i}} f\left(\mathbf{x} \, | \, y, \Theta_{y} \right) d\mathbf{x}\left\vert\!\vphantom{\int_{\Gamma_{i}} f\left(\mathbf{x} \, | \, y, \Theta_{y} \right) d\mathbf{x}S}\right. S \right]  \\ &= \int_{\Gamma_{i}} \text{E}\left[\,f\left(\mathbf{x} \, | \, y, \Theta_{y} \right) \, | \, S \right] d\mathbf{x}  \\ &= \int_{\Gamma_{i}}f\left(\mathbf{x} \, | \,y, S\right) d\mathbf{x}  \end{array} $$

(17) $$\begin{array}{*{20}l} &= \text{P}\left(\mathbf{X} \in \Gamma_{i} \, | \, y, S\right).  \end{array} $$

The second equality follows from Fubini’s theorem, and in the last equality, **X** is a random vector drawn from the density in the integrand of (16). We also have *f*(*y* | *S*)=E[*C*_*y*_ | *S*], which depends on the prior for **C** and is easily found, for instance, from (9) under Dirichlet posteriors. Comparing (3) and (15), observe that *f*(*y* | *S*) and *f*(**x** | *y*,*S*) play roles analogous to *f*(*y* | **c**) and *f*(**x** | *y*,*θ*_*y*_) in Bayes decision theory. We thus call *f*(**x** | *y*,*S*) the *effective class-y conditional density* or simply the *effective density*.

Whereas the BRE addresses overall classifier performance across the entire sample space, $\mathcal {X}$, we may also consider classification at a fixed point, $\mathbf {x} \in \mathcal {X}$. We define the *Bayesian conditional risk estimator* (BCRE) for class *i*∈{0,…,*M*−1} at point $\mathbf {x} \in \mathcal {X}$ to be the MMSE estimate of the conditional risk:

(18) $$\begin{array}{*{20}l} \widehat{R}(i, \mathbf{x}, S) &= \text{E}[R(i, \mathbf{x}, \mathbf{C}, \Theta) \, | \,S]  \\ &= \sum_{y = 0}^{M-1} \lambda(i, y) \text{E}[f(y \, | \, \mathbf{x}, \mathbf{C}, \Theta) \, | \, S ].  \end{array} $$

Again assuming {**X**,*Y*} and $\mathcal {S}$ are independent given **C** and *Θ*, and if we further assume **X** is independent from **C**, *Θ*, and $\mathcal {S}$, then,

$$\begin{array}{*{20}l} \text{E}[f(y \,| \, \mathbf{x}, \mathbf{C}, \Theta) \, | \, S ] &= \int f(y \, | \, \mathbf{x}, \mathbf{c}, \theta) f(\mathbf{c}, \theta \, | \, S) d\mathbf{c}d\theta  \\ &= \int f(y, \mathbf{c}, \theta \, | \, \mathbf{x}, S) d\mathbf{c}d\theta  \\ &= f(y \,| \, \mathbf{x}, S). \end{array} $$

Applying Bayes’ rule,

(19) $$\begin{array}{*{20}l} f(y \,| \, \mathbf{x}, S) &= \frac{f(y \,| \, S) f(\mathbf{x} \, | \, y, S)} {\sum_{y = 0}^{M-1} f(y \, | \,S) f(\mathbf{x} \, | \, y, S)},  \end{array} $$

and applying this to (18), we have

(20) $$\begin{array}{*{20}l} \widehat{R}(i, \mathbf{x}, S) &= \frac{\sum_{y = 0}^{M-1} \lambda(i, y)\, f(y \,| \, S) f(\mathbf{x} \, | \, y, S)} {\sum_{y = 0}^{M-1} f(y \,| \, S)\, f(\mathbf{x} \, | \, y, S)}.  \end{array} $$

This is analogous to (2) in Bayes decision theory. Furthermore, given a classifier *ψ*,

$$\begin{array}{*{20}l} \text{E}\left[ \widehat{R}(\psi(\mathbf{X}), \mathbf{X}, S)\left\vert\!\vphantom{\widehat{R}(\psi(\mathbf{X}), \mathbf{X}, S)S}\right. S \right] &= \sum_{i = 0}^{M-1} \int_{\Gamma_{i}} \widehat{R}(i, \mathbf{X}, S)\, f(\mathbf{x} \, | \, S) d\mathbf{x} \\ &= \widehat{R}(\psi, S), \end{array} $$

where $f(\mathbf {x} \, | \, S) = \sum _{y = 0}^{M-1} f(y \, | \, S) f(\mathbf {x} \, | \, y, S)$ is the marginal distribution of **x** given *S*. Hence, the BRE of *ψ* is the mean of the BCRE across the sample space.

For binary classification, $\widehat {\varepsilon }^{i, y}(\psi, S)$ has been solved in closed form as components of the BEE for both discrete models under arbitrary classifiers and Gaussian models under linear classifiers, so the BRE with an arbitrary loss function is available in closed form for both of these models. When closed-form solutions for $\widehat {\varepsilon }^{i, y}(\psi, S)$ are not available, from (17), $\widehat {\varepsilon }^{i, y}(\psi, S)$ may be approximated for all *i* and a given fixed *y* by drawing a large synthetic sample from *f*(**x** | *y*,*S*) and evaluating the proportion of points assigned class *i*. The final approximate BRE can be found by plugging the approximate $\widehat {\varepsilon }^{i, y}(\psi, S)$ for each *y* and *i* into (15).

A number of practical considerations for BEEs addressed under binary classification naturally carry over to multiple classes, including robustness to false modeling assumptions [9, 10] and a prior calibration method for microarray data analysis using features discarded by feature selection and a method-of-moments approach [21]. Furthermore, classical frequentist consistency holds for BREs on fixed distributions in the parameterized family owing to the convergence of posteriors in both the discrete and Gaussian models [12].

### Optimal Bayesian risk classification

We define the OBRC to minimize the BRE, that is,

(21) $$ \psi_{\text{OBRC}} = \text{arg} \inf_{\psi \in \mathcal{C}} \widehat{R}\left(\psi, S\right),   $$

where $\mathcal {C}$ is a family of classifiers. If $\mathcal {C}$ is the set of all classifiers with measurable decision regions, it can be shown that *ψ*_OBRC_ exists and is given for any $\mathbf {x} \in \mathcal {X}$ by

(22) $$ \psi_{\text{OBRC}}\left(\mathbf{x}\right) = \text{arg} \min_{i \in \{0, \ldots, M-1\}} \widehat{R}(i, \mathbf{x}, S).   $$

Analogously to the relationship between the BRE and expected risk, the OBRC has the same functional form as the BDR with *f*(*y* | *S*) substituted for the true class probability, *f*(*y* | **c**), and *f*(**x** | *y*,*S*) substituted for the true density, *f*(**x** | *y*,*θ*_*y*_), for all *y*. Closed-form OBRC are available for any model in which *f*(**x** | *y*,*S*) has been found, including discrete and Gaussian models [13]. A number of important properties also carry over, including invariance to invertible transformations, pointwise convergence to the Bayes classifier, and robustness to false modeling assumptions.

### Sample-conditioned MSE of risk estimation

In a typical small-sample classification scenario, a classifier is trained from data and a risk estimate found for the true risk of this classifier. A key question arises: How close is the risk estimate to the actual risk? A Bayesian approach answers this question with the sample-conditioned MSE of the BRE relative to the true expected risk:

(23) $$\begin{array}{*{20}l} \text{MSE} (\widehat{R }(\psi, S) \, | \, S) &= \text{E}\left[ \left(R\left(\psi, \mathbf{C}, \Theta \right) -\widehat{R }(\psi, S)\right)^{2}\bigg| {S} \right]  \\ &=\text{Var}\left(R(\psi, \mathbf{C}, \Theta) \, | \,S\right).  \end{array} $$

This MSE is precisely the quantity that the BRE minimizes, and it quantifies the accuracy of $\widehat {R}$ as an estimator of *R*, conditioned on the actual sample in hand. Thanks to posterior independence between **C** and *Θ*, it can be decomposed:

(24) $$\begin{array}{*{20}l} &\text{MSE}(\widehat{R }(\psi, S) \, | \, S)  \\ &= \left(\sum_{y = 0}^{M-1} \sum_{z = 0}^{M-1} \sum_{i = 0}^{M-1} \sum_{j = 0}^{M-1} \lambda(i, y) \lambda(j, z) \text{E}\left[ C_{y} C_{z} \, | \, S \right] \right.\\ &\qquad \times\left. \text{E}\left[ \varepsilon^{i, y}(\psi, \Theta_{y}) \varepsilon^{j, z}(\psi, \Theta_{z}) \,| \, S \right] {\vphantom{\sum_{y = 0}^{M-1}}}\right) - (\widehat{R}(\psi, S))^{2},  \end{array} $$

where we have applied (3) in (23) and noted $\text {E}\left [\,f(y \, | \, \mathbf {C})\right. \left. f\!(z \, | \, \mathbf {C}) \, | \, S{\vphantom {\sum }} \right ] = \text {E}\left [ C_{y} C_{z} \, | \, S \right ]$. Second-order moments of *C*_*y*_ depend on our prior for **C** and can be found, for instance, from (10) and (11) under Dirichlet posteriors. Hence, evaluating the conditional MSE of the BRE boils down to evaluating the BRE itself, $\widehat {R}(\psi, S)$, and evaluating expressions of the form E[*ε* ^*i*,*y*^(*ψ*,*Θ*_*y*_)*ε* ^*j*,*z*^(*ψ*,*Θ*_*z*_) | *S*]. Furthermore, if we additionally assume *Θ*_0_,…,*Θ*_*M*−1_ are pairwise independent, then when *y*≠*z*,

(25) $$\begin{array}{*{20}l} \text{E} \left[ \varepsilon^{i, y}(\psi, \Theta_{y}) \varepsilon^{j, z}(\psi, \Theta_{z}) \, | \, S \right] = \,\widehat{\varepsilon}^{i, y}(\psi, S) \widehat{\varepsilon}^{j, z}(\psi, S),  \end{array} $$

where $\widehat {\varepsilon }^{i, y}(\psi, S)$, given in (16), is a component of the BRE. The conditional MSE of an arbitrary risk estimate, $\widehat {R }_{\bullet }(\psi, S)$, is also of interest and may be easily found from the BRE and the MSE of the BRE:

(26) $$\begin{array}{*{20}l} & \text{MSE}(\widehat{R }_{\bullet }(\psi, S) \,| \, S)  \\ &= \text{E}\left[\left(R\left(\psi, \mathbf{C}, \Theta \right) -\widehat{R }_{\bullet }(\psi, S)\right)^{2}\bigg| {S} \right]  \\ & =\text{MSE}(\widehat{R }(\psi, S) \, | \,S) + (\widehat{R }(\psi, S)-\widehat{R }_{\bullet }(\psi, S))^{2}.  \end{array} $$

In this form, the optimality of the BRE is clear.

For binary classification with zero-one loss, the sample-conditioned MSE of the BRE converges to zero almost surely as sample size increases, for both discrete models under arbitrary classifiers and Gaussian models with independent covariances under linear classifiers [12]. Closed-form expressions for the MSE are available in these models. In this work, we extend this to multi-class discrimination under discrete models and binary linear classification under homoscedastic Gaussian models. For cases where closed-form solutions are unavailable, in the next section, we present a method to approximate the MSE.

### Efficient computation

The following new interpretation for $\text {E} \left [ \varepsilon ^{i, y}(\psi, \Theta _{y}) \right. \left. \varepsilon ^{j, z} (\psi, \Theta _{z}) \, | \, S \vphantom {\sum {}}\right ]$ is useful in both deriving analytic forms for and approximating the MSE. From (4),

(27) $$\begin{array}{*{20}l} &\text{E}\left[ \varepsilon^{i, y}(\psi, \Theta_{y}) \varepsilon^{j, z}(\psi, \Theta_{z}) \, | \, S \right]  \\ &= \int_{\mathcal{T}} \int_{\Gamma_{i}} f(\mathbf{x} \, | \, y, \theta_{y}) d\mathbf{x} \int_{\Gamma_{j}} f(\mathbf{w}\,| \, z, \theta_{z}) d\mathbf{w} f(\theta \, | \, S)d\theta  \\ &= \int_{\Gamma_{i}} \int_{\Gamma_{j}} \int_{\mathcal{T}} f(\mathbf{x} \, | \, y, \theta_{y}) f(\mathbf{w} \, | \, z, \theta_{z}) f(\theta \, | \, S) d\theta d\mathbf{w} d\mathbf{x}, \end{array} $$

where we have again applied Fubini’s theorem. Further, we may write

(28) $$\begin{array}{*{20}l} &\text{E}\left[ \varepsilon^{i, y}(\psi, \Theta_{y}) \varepsilon^{j, z}(\psi, \Theta_{z}) \,| \, S \right]  \\ &\qquad\qquad= \int_{\Gamma_{i}} \int_{\Gamma_{j}} f(\mathbf{x}, \mathbf{w} \, | \,y, z, S) d\mathbf{w} d\mathbf{x}  \end{array} $$

(29) $$\begin{array}{*{20}l} &\qquad\qquad= \text{P}\left(\mathbf{X} \in \Gamma_{i} \cap \mathbf{W} \in \Gamma_{j} \, | \,y, z, S\right),  \end{array} $$

where **X** and **W** are random vectors drawn from an *effective joint density*, defined using similar independence assumptions as in (14):

(30) $$\begin{array}{*{20}l} f(\mathbf{x}, \mathbf{w} \,| \, y, z, S) &= \int f(\mathbf{x} \, | \, y, \theta_{y}) f(\mathbf{w} \, | \, z, \theta_{z}) f(\theta \, | \, S) d\theta.  \end{array} $$

The marginal densities of **X** and **W** under *f*(**x**,**w** | *y*,*z*,*S*) are precisely the effective density, i.e.,

$$\begin{array}{*{20}l} &\int_{\mathcal{X}} f(\mathbf{x}, \mathbf{w} \,| \, y, z, S) d\mathbf{w} \\ &= \int_{\mathcal{X}} \int_{\mathcal{T}} f(\mathbf{x} \,| \, y, \theta_{y}) f(\mathbf{w} \, | \, z, \theta_{z}) f(\theta \, | \, S) d\theta d\mathbf{w} \\ &= \int_{\mathcal{T}} f(\mathbf{x} \, | \, y, \theta_{y}) \int_{\mathcal{X}} f(\mathbf{w} \, | \, z, \theta_{z}) d\mathbf{w} f(\theta \, | \,S) d\theta \\ &= \int_{\mathcal{T}_{y}} f(\mathbf{x} \, | \, y, \theta_{y}) f(\theta_{y} \, | \, S) d\theta_{y} \\ &= f(\mathbf{x} \, | \, y, S), \end{array} $$

where *f*(*θ*_*y*_ | *S*) is the marginal posterior density of *Θ*_*y*_. Further, we have an *effective conditional density* of **W** given **X**,

$$\begin{array}{*{20}l} f(\mathbf{w} \,| \, \mathbf{x}, y, z, S) &= \frac{f(\mathbf{x}, \mathbf{w} \, | \, y, z, S)}{f(\mathbf{x} \, | \, y, S)} \\ &= \int f(\mathbf{w} \, | \, z, \theta_{z}) \frac{f(\mathbf{x} \,| \, y, \theta_{y}) f(\theta \, | \, S)} {\int f(\mathbf{x} \, | \, y, \theta_{y}^{\prime}) f(\theta_{y}^{\prime} \, | \, S) d\theta_{y}^{\prime}} d\theta \\ &= \int f(\mathbf{w} \, | \, z, \theta_{z}) f(\theta \, | \, S \cup \{\mathbf{x}, y\}) d\theta \\ &= f(\mathbf{w} \, | \, z, S \cup \{\mathbf{x}, y\}), \end{array} $$

where we have used the fact that the fractional term in the integrand of the second equality is of the same form as the posterior defined in (8), updated with a new independent sample point with feature vector **x** and class *y*. Hence, the effective joint density may be easily found, once the effective density is known. Furthermore, from (29), we may approximate E[*ε* ^*i*,*y*^(*ψ*,*Θ*_*y*_)*ε* ^*j*,*z*^(*ψ*,*Θ*_*z*_) | *S*] by drawing a large synthetic sample from *f*(**x** | *y*,*S*), drawing a single point, **w**, from the effective conditional density *f*(**w** | *z*,*S*∪{**x**,*y*}) for each **x**, and evaluating the proportion of pairs, (**x**,**w**), for which **x**∈*Γ*_*i*_ and **w**∈*Γ*_*j*_. Additionally, since **x** is marginally governed by the effective density, from (17) we may approximate $\widehat {\varepsilon }^{i,y}(\psi, S)$ by evaluating the proportion of **x** in *Γ*_*i*_.

Evaluating the OBRC, BRE, and conditional MSE requires obtaining E[*C*_*y*_ | *S*], ${\text {E}[C_{y}^{2}} \, | \, S]$ and E[*C*_*y*_ *C*_*z*_ | *S*] based on the posterior for **C** and finding the effective density, *f*(**x** | *y*,*S*), and the effective joint density, *f*(**x**,**w** | *y*,*z*,*S*), based on the posterior for *Θ*. At a fixed point, **x**, one may then evaluate the posterior probability of each class, *f*(*y* | **x**,*S*), from (19) and the BCRE from (20). The OBRC is then found from (22) or, equivalently, by choosing the class, *i*, that minimizes $\sum _{y = 0}^{M-1} \lambda (i, y) \text {E}[C_{y} \, | \, S] f(\mathbf {x} \, | \, y, S)$. For any classifier, the BRE is given by (15) with $\widehat {\varepsilon }^{i,y}(\psi, S)$ given by (16) (or equivalently (17)) using the effective density, *f*(**x** | *y*,*S*). The MSE of the BRE is then given by (24), where E[*ε* ^*i*,*y*^(*Θ*_*y*_)*ε* ^*j*,*z*^(*Θ*_*z*_) | *S*] is given by (25) when *Θ*_0_,…,*Θ*_*M*−1_ are pairwise independent and *y*≠*z*, and E[*ε* ^*i*,*y*^(*Θ*_*y*_)*ε* ^*j*,*z*^(*Θ*_*z*_) | *S*] is otherwise found from (28) (or equivalently (29)) using the effective joint density, *f*(**x**,**w** | *y*,*z*,*S*). The MSE of an arbitrary risk estimator can also be found from (26) using the BRE and the MSE for the BRE. We summarize these tools for several discrete and Gaussian models in Appendices Appendix 1: Discrete models, Appendix 2: Gaussian models, and Appendix 3: Effective joint density lemma by providing the effective density, the effective joint density (or a related density), $\widehat {\varepsilon }^{i,y}(\psi, S)$, and E[*ε* ^*i*,*y*^(*Θ*_*y*_)*ε* ^*j*,*z*^(*Θ*_*z*_) | *S*].

## Simulation setup and results

In the this section, we examine several synthetic data simulations, where random distributions and samples are generated from a low-information prior, and demonstrate the performance gain and optimality of Bayesian methods within the Bayesian framework. We also examine performance with informed priors in two real datasets.

### Classification rules

We consider five classification rules: OBRC, linear discriminant analysis (LDA), quadratic discriminant analysis (QDA), linear support vector machine (L-SVM), and radial basis function SVM (RBF-SVM). We will implement OBRC under Gaussian models. We used built-in MATLAB functions to implement LDA and QDA. For a collection of binary-labeled training sample points, an SVM classifier finds a maximal margin hyperplane based on a well-behaved optimization objective function and a set of constraints. When the data are not perfectly linearly separable, introduction of slack variables in the optimization procedure leads to *soft margin* classifiers for which mislabeled sample points are allowed. The resulting hyperplane in the feature space is called L-SVM. Alternatively, the underlying feature space can be transformed to a higher dimensional space where the data becomes linearly separable. The equivalent classifier back in the original feature space will generally be non-linear [22, 23]. When the kernel function is a Gaussian radial basis function, we call the corresponding classifier RBF-SVM. We used the package LIBSVM, which, by default, implements a *one-versus-one* approach for multi-class classification [24]. Since SVM classifiers optimize relative to their own objective function (for example, hinge loss), rather than expected risk, we exclude them from our analysis when using a non-zero-one loss function.

For all classification rules, we calculate the true risk defined in (3) and (4). We find the exact value if a formula is available; otherwise, we use a test sample of at least 10,000 points generated from the true feature-label distributions, stratified relative to the true class prior probabilities. This will yield an approximation of the true risk with RMS $\leq 1/\sqrt {4 \times 10,000} = 0.005$ [8].

### Risk estimation rules

We consider four risk estimation methods: BRE, 10-fold cross-validation (CV), leave-one-out (LOO), and 0.632 bootstrap (boot). When we do not have closed-form formulae for calculating the BRE, we approximate it by drawing a sample of 1,000,000 points from the effective density of each class. In CV, the training data, *S*, is randomly partitioned into 10 stratified folds, *S* ^(*i*)^ for *i*=1,2,…,10. Each fold, in turn, is held out of the classifier design step as the test set, and a surrogate classifier is designed on the remaining folds, *S*∖*S* ^(*i*)^, as the training set. The risk of each surrogate classifier is estimated using *S* ^(*i*)^. The resulting risk values from all surrogate classifiers are then averaged to get the CV estimate. To reduce “internal variance” arising from random selection of the partitions, we average the CV estimates over 10 repetitions (10 randomly generated partitions over *S*). If the number of folds equals the sample size, *n*, then each fold consists of a single point and we get the LOO risk estimation.

Bootstrap risk estimators are calculated using bootstrap samples of size *n*, where in each bootstrap sample, points are drawn, with replacement, from the original training dataset. A surrogate classifier is designed on the bootstrap sample and its risk estimated using sample points left out of the bootstrap sample. The basic bootstrap estimator is the expectation of this risk with respect to the bootstrap sampling distribution. The expectation is usually approximated by Monte Carlo repetitions (100 in our simulations) over a number of independent bootstrap samples. It is known that this estimate is high biased. To reduce bias, the 0.632 bootstrap reports a linear combination of this estimate, with weight 0.632, and the low-biased resubstitution risk estimate, with weight 0.368 [25–27].

Under linear classification, the sample-conditioned MSE from (24) is found analytically by evaluating E[*ε* ^*i*,*y*^(*Θ*_*y*_)*ε* ^*j*,*y*^(*Θ*_*y*_) | *S*] from (52), plugging in the appropriate values for *k* and *γ* ^2^ depending on the covariance model, and E[*ε* ^*i*,*y*^(*Θ*_*y*_)*ε* ^*j*,*z*^(*Θ*_*z*_) | *S*] for *z*≠*y* are found via (25) for independent and (53) for homoscedastic covariance models, plugging in appropriate values for *k* and *γ* ^2^. When analytic forms are not available, the sample-conditioned MSE is approximated as follows. In independent covariance models, for each sample point generated to approximate the BRE, we draw a single point from the effective conditional density with *y*=*z*, giving 1,000,000 sample point pairs to approximate E[*ε* ^*i*,*y*^(*Θ*_*y*_)*ε* ^*j*,*y*^(*Θ*_*y*_) | *S*] for each *y*. In homoscedastic covariance models, to find the BRE, we have 1,000,000 points available from the effective density for each *y*. We generate an additional 1,000,000×(*M*−1) synthetic points for each *y*, thus allocating 1,000,000 synthetic points for each combination of *y* and *z*. For each of these points, we draw a single point from the effective conditional density of a class-*z* point given a class-*y* point. For each *y* and *z*, the corresponding 1,000,000 point pairs are used to approximate E[*ε* ^*i*,*y*^(*Θ*_*y*_)*ε* ^*j*,*z*^(*Θ*_*z*_) | *S*].

### Synthetic data

In synthetic data simulations, we assume all classes are equally likely and that the data is stratified, giving an equal number of sample points from each class. We further assume Gaussian feature-label distributions. Table 1 lists all models and prior distributions used. We implement both a low number of features (*D*=2) and a high number of features (*D*=20), with independent arbitrary, homoscedastic arbitrary, and independent identity covariance priors. Under each type of prior, we consider classification under a non-zero-one loss function for binary classification and a zero-one loss function for multiple classes. For each prior model and a fixed sample size, we evaluate classification performance in a Monte Carlo estimation loop with 10,000 iterations. In each iteration, we follow a two-step procedure for sample generation: (1) generate random feature-label distribution parameters from the prior (each serving as the true underlying feature-label distribution) and (2) generate a random sample of size *n* from this fixed feature-label distribution. The generated random sample is used to train classifiers and evaluate their true risk. In the non-zero-one loss case, we also estimate risk and evaluate its accuracy using the performance metrics discussed earlier. We vary the sample size throughout and analyze its effect on performance.

**Table 1:** Synthetic data classification settings and prior models

	*D*	*M*	*ν*_0_,…,*ν*_*M*−1_	**m**_0_,…,**m**_*M*−1_	*κ*_*y*_ (*k*_*y*_)	$\frac {\mathbf {S}_{y}}{k_{y} - 2}$	Prior (cov.)	*λ*
Model 1	2	2	12, 2	$\left [\begin {array}{l} 0 \\ 0 \end {array}\right ], \left [\begin {array}{l} 0.5 \\ 0.5 \end {array}\right ]$	6 (5)	0.3 *I*_2_	Indep. arbit.	$\left [\begin {array}{ll} 0 & 2 \\ 1 & 0 \end {array}\right ]$
Model 2	2	2	12, 2	$\left [\begin {array}{l} 0 \\ 0 \end {array}\right ], \left [\begin {array}{l} 0.5 \\ 0.5 \end {array}\right ]$	6 (5)	0.3 *I*_2_	Homo. arbit.	$\left [\begin {array}{ll} 0 & 2 \\ 1 & 0 \end {array}\right ]$
Model 3	2	5	12, 2, 2, 2, 2	$\left [\begin {array}{l} 0 \\ 0 \end {array}\right ], \left [\begin {array}{l} 1 \\ 1 \end {array}\right ], \left [\begin {array}{l} -1 \\ -1 \end {array}\right ], \left [\begin {array}{l} 1 \\ -1 \end {array}\right ], \left [\begin {array}{l} -1 \\ 1 \end {array}\right ]$	6 (5)	0.3 *I*_2_	Indep. arbit.	0–1 loss
Model 4	2	5	12, 2, 2, 2, 2	$\left [\begin {array}{l} 0 \\ 0 \end {array}\right ], \left [\begin {array}{l} 1 \\ 1 \end {array}\right ], \left [\begin {array}{l} -1 \\ -1 \end {array}\right ], \left [\begin {array}{l} 1 \\ -1 \end {array}\right ], \left [\begin {array}{l} -1 \\ 1 \end {array}\right ]$	6 (5)	0.3 *I*_2_	Homo. arbit.	0–1 loss
Model 5	20	2	12, 2	0_20_,(0.05)_20_	−20.65 (5)	0.3 *I*_2_	Indep. iden.	$\left [\begin {array}{ll} 0 & 2 \\ 1 & 0 \end {array}\right ]$
Model 6	20	2	20, 20	0_20_,0_20_	−20.65 (5)	0.3 *I*_20_	Indep. iden.	$\left [\begin {array}{ll} 0 & 2 \\ 1 & 0 \end {array}\right ]$
Model 7	20	5	12, 2, 2, 2, 2	0_20_,(0.1)_20_,(−0.1)_20_,	−20.65 (5)	0.3 *I*_20_	Indep. iden.	0–1 loss
				$\left [\begin {array}{l} {(0.1)}_{10} \\ {(-0.1)}_{10} \end {array}\right ], \left [\begin {array}{l} {(-0.1)}_{10} \\ {(0.1)}_{10} \end {array}\right ]$				
Model 8	20	5	20, 20, 20, 20, 20	0_20_,0_20_,0_20_,0_20_,0_20_	−20.65 (5)	0.3 *I*_20_	Indep. iden.	0–1 loss

0_*k*_ and (*a*)_*k*_ represent all-zero and all-*a* column vectors of length *k*, respectively

### Real data

We consider two real datasets. The first is a breast cancer dataset containing 295 sample points [28], which will be used to demonstrate binary classification under a non-zero-one loss function. The second is composed of five different cancer types from The Cancer Genome Atlas (TCGA) project, which demonstrates multi-class classification under zero-one loss.

In all real-data simulations, we assume that *c*_*y*_ is known and equal to the proportion of class-*y* sample points in the whole dataset. We form a Monte Carlo estimation loop to evaluate classification and risk estimation, where we iterate 1000 times with the breast cancer dataset and 10,000 times with the TCGA dataset. In each iteration, we obtain a stratified training sample of size *n*, i.e., we select a subset of the original dataset, keeping the proportion of points in class *y* as close as possible to *c*_*y*_ for every *y*. We use these training points to design several classifiers, while the remaining sample points are used as holdout data to approximate the true risk of each designed classifier. For the breast cancer dataset, we also use the training data to estimate risk and find the sample-conditioned MSE of the BRE. We vary sample size and analyze its effect on performance.

To implement Bayesian methods, we assume Gaussian distributions with arbitrary independent covariances in all real-data simulations. We calibrate hyperparameters, defined in Appendix Appendix 2: Gaussian models, using a variant of the method-of-moments approach presented in [21]. In particular, we construct a calibration dataset from features not used to train the classifier and set *ν*_*y*_=*s*_*y*_/*t*_*y*_, $\kappa _{y} = 2({s_{y}^{2}}/u_{y})\,+\,D\,+\,3$, **m**_*y*_=[*m*_*y*_,…,*m*_*y*_], and **S**_*y*_=(*κ*_*y*_−*D*−1)*s*_*y*_ **I**_*D*_, where *m*_*y*_ is the mean of the means of features among class-*y* points of the calibration dataset, and *s*_*y*_ is the mean of the variances of features in class *y*. *t*_*y*_ is the variance of the means of features in class *y*, where the 10 % of the means with the largest absolute value are discarded. Likewise, *u*_*y*_ is the variance of the variances of features in class *y*, where the 10 % of the variances with the largest value are discarded.

In the breast cancer data, 180 patients are assigned to class 0 (good prognosis) and 115 to class 1 (bad prognosis) in a 70-feature prognosis profile. A correct prognosis is associated with 0 loss, wrongly declaring a good prognosis incurs a loss of 1, and wrongly declaring a bad prognosis incurs a loss of 2. We use pre-selected features for classifier training, originally published in [29]. When *D*=2, these features are CENPA and BBC3, and when *D*=5, we also add CFFM4, TGFB3, and DKFZP564D0462. Rather than discard the 70 − *D* features not used for classification, we use these features to calibrate priors using the method-of-moments approach described above.

For our second dataset, we downloaded level-3 microarray data from the TCGA data portal for five different kinds of cancers: breast invasive carcinoma (BRCA) with 593 sample points, colon adenocarcinoma (COAD) with 174 sample points, kidney renal clear cell carcinoma (KIRC) with 72 sample points, lung squamous cell carcinoma (LUSC) with 155 sample points, and ovarian serous cystadenocarcinoma (OV) with 562 sample points. We pooled all the sample points into a single dataset, removed features with missing values in any cancer type (17,016 features remained out of 17,814), and quantile-normalized the data with the median of the ranked values. We pre-select features for classifier training and prior calibration using the full dataset and one of two methods, which both operate in two phases: in phase 1, we pass *D*+100 features, and in phase 2, we select *D* features from those passing phase 1. The *D* features passing both phases are used for classifier training, and the features passing phase 1 but not phase 2 are used for prior calibration. The first feature selection method (FS-1) passes features that minimize a score evaluating separation between classes in phase 1 and selects features that minimize a score evaluating Gaussianity of the classes in phase 2. To evaluate separation between classes in phase 1, for each pair of classes, we obtain *t-*test *p*-values for each feature and rank these across all features, low *p*-values being assigned a lower rank, and finally, we report the rank product score for each feature over all 10 pairs of classes. To evaluate Gaussianity in phase 2, for each class, we rank Shapiro-Wilk test *p*-values across all features passing phase 1, high *p*-values being assigned a lower rank, and report the rank product score for each feature across all five classes. The second feature selection method (FS-2) passes features minimizing the rank product score from Shapiro-Wilk tests applied to all 17,016 features in phase 1, and in phase 2, we select *D* features from those passing phase 1 using sequential forward search (SFS) with LDA classification and resubstitution risk as the optimization criterion.

### Discussion

**Fig. 1:**
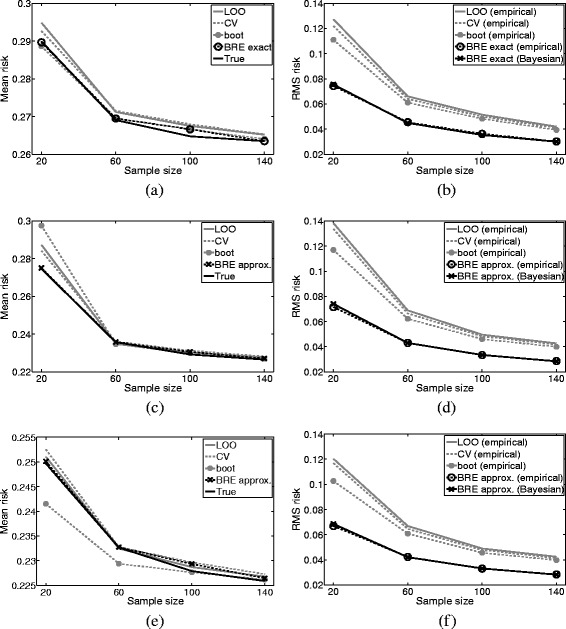
Mean risks and RMS for model 1, three classification rules (LDA, QDA, and OBRC), and all risk estimators. **a** Mean risk under LDA; **b** RMS risk under LDA; **c** mean risk under QDA; **d** RMS risk under QDA; **e** mean risk under OBRC; **f** RMS risk under OBRC

Models 1 and 2 focus on the effect of risk on classification and risk estimation performance. In Fig. 1, we evaluate the performance of risk estimators and classifiers under model 1. Graphs in the left column present the mean, averaged over all 10,000 sample realizations, of the true risk and all risk estimators considered for LDA, QDA, and OBRC classification. Note for small samples of size *n*=20 and LDA or QDA classification, surrogate classifiers in the bootstrap risk estimator are occasionally undefined depending on the realized bootstrap sample. These events are thrown out so that only a subset of the original 10,000 sample realizations are used to approximate the mean bootstrap risk estimator. The graphs on the right column of Fig. 1 present the square root of the mean, averaged over all sample realizations, of the square difference between the true risk and each risk estimator, which we call the *empirical RMS*. The square root of the mean, averaged over all sample realizations, of the sample-conditioned MSE of the BRE from (24), which we call the *Bayesian RMS*, is also shown.

**Fig. 2:**
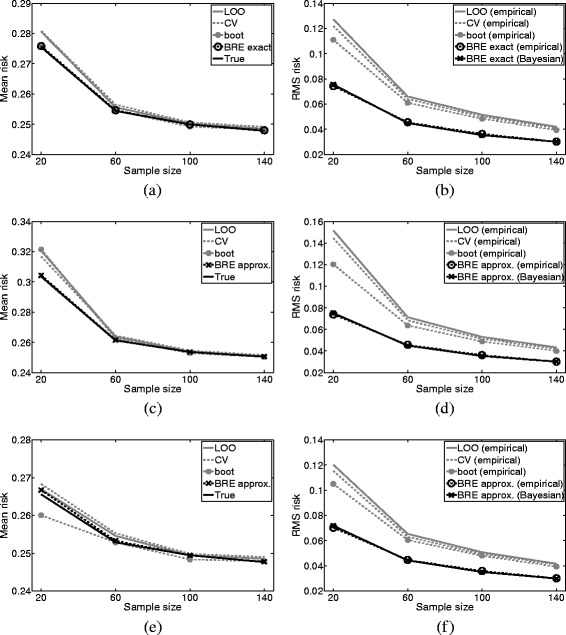
Mean risks and RMS for model 2, three classification rules (LDA, QDA, and OBRC), and all risk estimators. **a** Mean risk under LDA; **b** RMS risk under LDA; **c** mean risk under QDA; **d** RMS risk under QDA; **e** mean risk under OBRC; **f** RMS risk under OBRC

The BRE is an unbiased estimator, so the mean true risk and mean BRE curves should be aligned with enough iterations, which is observed. The empirical and Bayesian RMS both approximate the unconditional RMS so that these curves should also be aligned with enough iterations, as observed. Furthermore, the BRE is theoretically optimal in both the sample-conditioned and unconditioned RMS, and as expected, the empirical and Bayesian RMS curves for BRE under each classification rule outperform all other risk estimation rules. Thus, the BRE yields a significant improvement over classical risk estimators in terms of both bias and RMS performance within the Bayesian model. If we compare classification rules, the RMS of BRE is consistently lower for OBRC than LDA and QDA, although there is no theoretical guarantee for this. Similar curves for model 2 are provided in Fig. 2.

**Fig. 3:**
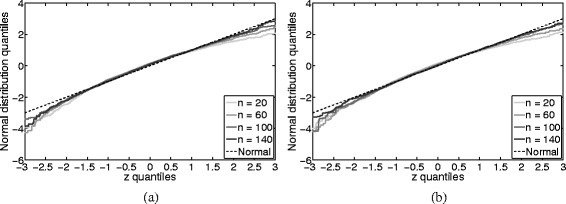
Q-Q plots of *Z* under OBRC and BRE. **a** Model 1; **b** model 2

To illustrate how the sample-conditioned MSE may be used to assess the accuracy of a risk estimate, suppose that we have observed a sample, trained a classifier, and obtained the BRE and the MSE of the BRE. For this fixed sample, but random parameters in the Bayesian model, the true risk has a mean equal to the BRE and a variance equal to the sample-conditioned MSE so that the random variable $Z = (\widehat {R} - R)/\text {RMS}(\widehat {R}|S)$ must have zero mean and unit variance. This holds for any classification rule. In Fig. 3, we present quantile-quantile (Q-Q) plots of the sample quantiles of *Z* versus theoretical quantiles from a standard normal distribution. Figure 3a provides Q-Q plots with realizations of *Z* taken under OBRC classification and BRE risk estimation in model 1 with various sample sizes, along with a 45° reference line, and Fig. 3 b provides similar graphs for model 2. Observe that *Z* appears approximately standard normal, particularly under large sample sizes. Under smaller samples, *Z* appears more positively skewed but has approximately zero mean and unit variance. Q-Q plots for other classifiers are similar.

**Fig. 4:**
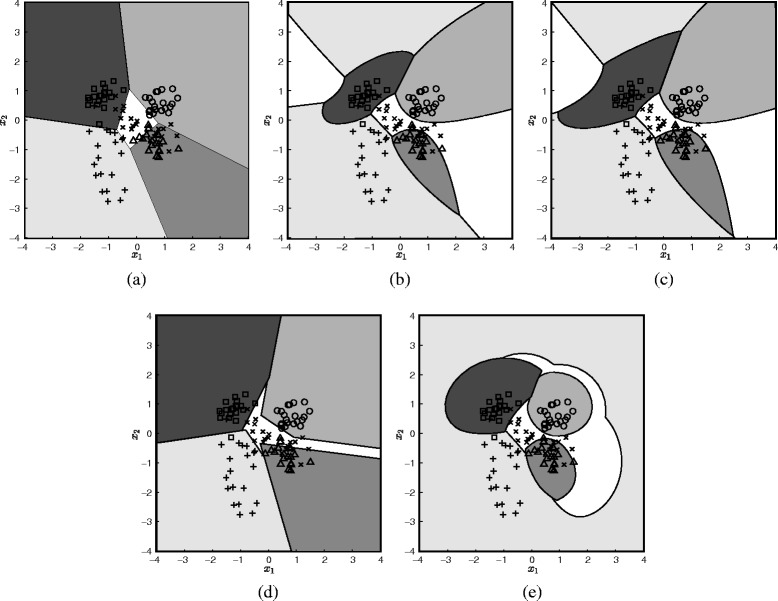
Example decision boundaries for model 3 with multi-class classification. **a** LDA; **b** QDA; **c** OBRC; **d** L-SVM; **e** RBF-SVM

**Fig. 5:**
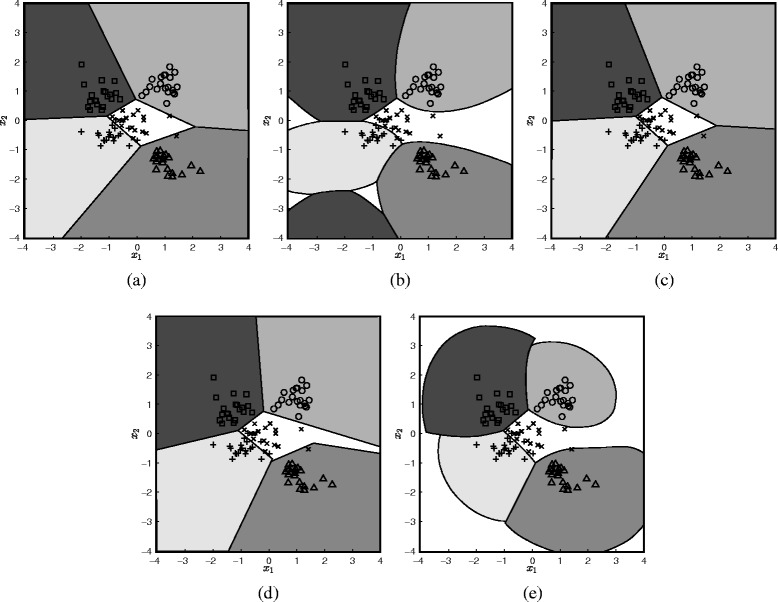
Example decision boundaries for model 4 with multi-class classification. **a** LDA; **b** QDA; **c** OBRC; **d** L-SVM; **e** RBF-SVM

In Figs. 4 and 5, we provide examples of decision boundaries for models 3 and 4, respectively, which focus on the effect of multiple classes in two dimensions. Under model 3, where we assume independent covariances, the decision boundaries of OBRC are most similar to QDA, although they are in general of a polynomial order. Under model 4, where we assume homoscedastic covariances, OBRC is most similar to LDA, although the decision boundaries are not necessarily linear.

**Fig. 6:**
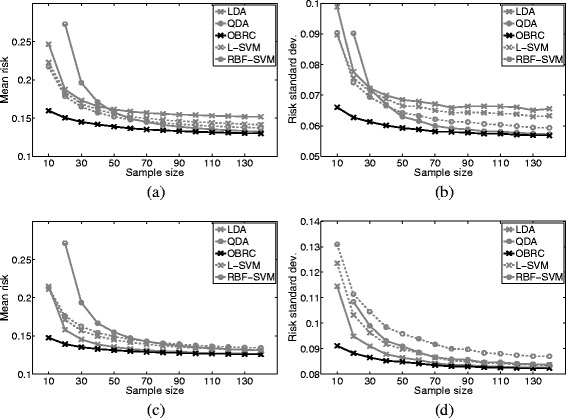
True risk statistics for models 3 and 4 and five classification rules (LDA, QDA, OBRC, L-SVM, and RBF-SVM). **a** Model 3, mean; **b** model 3, standard deviation; **c** model 4, mean; **d** model 4, standard deviation

In Fig. 6, we present the mean and standard deviation of the true risk with respect to all sample realizations as a function of sample size for models 3 and 4. OBRC outperforms all other classification rules with respect to mean risk, as it must, since the OBRC is defined to minimize mean risk. Although there is no guarantee that OBRC should minimize risk variance, in these examples, the risk variance is lower than in all other classification rules. The performance gain is particularly significant for small samples. Consider Figs. 6 a and 6 b, where we observe that, at a sample size of 10, the risk of OBRC has a mean of about 0.16 and standard deviation of about 0.065, whereas the risk of the next best classifier, RBF-SVM, has a mean of about 0.22 and standard deviation of about 0.09.

**Fig. 7:**
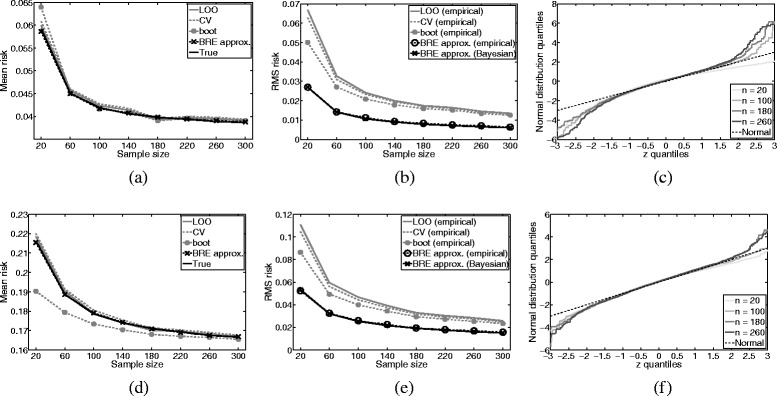
Mean risks, RMS, and Q-Q plots of *Z* for models 5 and 6, OBRC classification, and all risk estimators. **a** Mean risk under model 5; **b** RMS risk under model 5; **c** Q-Q plots of *Z* under model 5; **d** mean risk under model 6; **e** RMS risk under model 6; **f** Q-Q plots of *Z* under model 6

Figure 7 provides the performance of risk estimators under OBRC classification in models 5 and 6, demonstrating performance in 20 dimensions with independent scaled identity covariance priors. Settings in model 5 are designed to produce a low mean risk and model 6 a high mean risk. Graphs in the left column present the mean true risk, averaged over all 10,000 sample realizations; the center column presents empirical and Bayesian RMS curves; and the right column presents Q-Q plots of *Z*. As in Figs. 1 and 2, the BRE appears unbiased, the empirical and Bayesian RMS curves are aligned, and the RMS curves are optimal. From the Q-Q plots, the distribution of *Z* appears to be skinny-tailed even under large *n*, although it is approximately zero mean and unit variance.

**Fig. 8:**
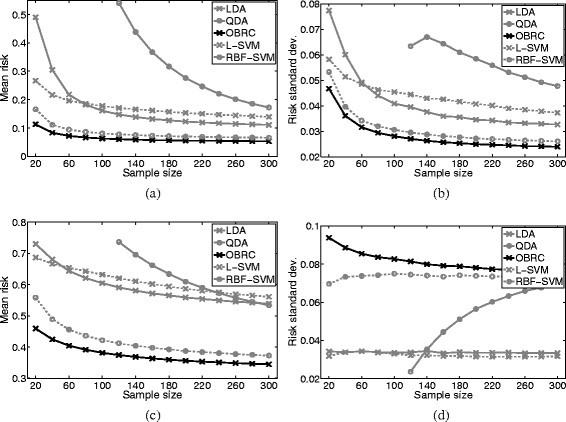
True risk statistics for models 7 and 8 and five classification rules (LDA, QDA, OBRC, L-SVM, and RBF-SVM). **a** Model 7, mean; **b** model 7, standard deviation; **c** model 8, mean; **d** model 8, standard deviation

In Fig. 8, we present the mean and standard deviation of the true risk of all classifiers as a function of sample size for models 7 and 8, where model 7 is designed to produce a low mean risk and model 8 a high mean risk. OBRC again outperforms all other classification rules with respect to mean risk, as it should. There is no guarantee that OBRC should minimize risk variance, and although risk variance is lowest for OBRC in Fig. 8 b, in Fig. 8 d it is actually highest. Performance gain is particularly significant for small samples.

**Fig. 9:**
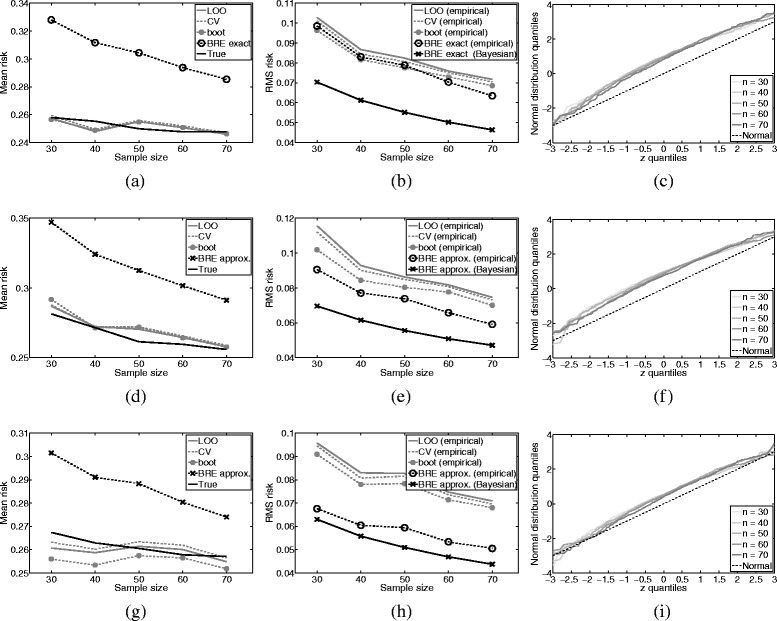
Mean risks, RMS, and Q–Q plots of *Z* for the breast cancer dataset, *D*=2, three classification rules (LDA, QDA, and OBRC), and all risk estimators. **a** Mean risk under LDA; **b** RMS risk under LDA; **c** Q–Q plots of *Z* under LDA; **d** mean risk under QDA; **e** RMS risk under QDA; **f** Q–Q plots of *Z* under QDA; **g** mean risk under OBRC; **h** RMS risk under OBRC; **i** Q–Q plots of *Z* under OBRC

**Fig. 10:**
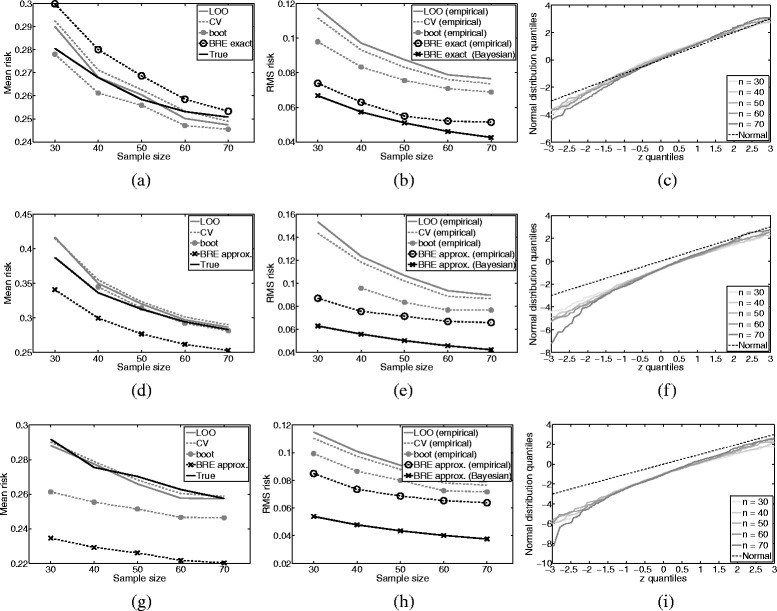
Mean risks, RMS and Q–Q plots of *Z* for the breast cancer dataset, *D*=5, three classification rules (LDA, QDA, and OBRC), and all risk estimators. **a** Mean risk under LDA; **b** RMS risk under LDA; **c** Q-Q plots of *Z* under LDA; **d** mean risk under QDA; **e** RMS risk under QDA; **f** Q–Q plots of *Z* under QDA; **g** mean risk under OBRC; **h** RMS risk under OBRC; **i** Q–Q plots of *Z* under OBRC

In Figs. 9 and 10, we evaluate the performance of risk estimators and classifiers under the breast cancer dataset for *D*=2 and *D*=5, respectively. Graphs in the left column present the mean true risk and mean risk estimates, graphs in the center column present the empirical RMS of all risk estimates and the Bayesian RMS for the BRE, and graphs in the right column present the Q-Q plots of *Z* for various sample sizes. LDA, QDA, and OBRC are presented in the top, center, and bottom rows, respectively. Although the BRE is theoretically unbiased and minimizes RMS when averaged across random distributions in the uncertainty class, when applied to a specific dataset or distribution, we now observe a bias (in the left column) and a discrepancy between the empirical and Bayesian RMS (in the center column). In particular, for all classifiers under *D*=2 and for LDA under *D*=5, we observe a high bias, for QDA and OBRC under *D*=5, we observe a low bias, and in all cases, the Bayesian RMS lies below the empirical RMS. That being said, the empirical RMS still outperforms that of distribution-free resampling error estimators (LOO, CV, and boot). Although resampling estimators are nearly unbiased, they suffer from such large variance under small samples that the BRE, despite imperfections in the Gaussianity assumption and prior construction method, may still outperform in practice thanks to optimization. Turning to classifier performance, in these simulations, LDA appears to outperform QDA and OBRC with independent arbitrary covariances. Keep in mind that Bayesian methods are not guaranteed to be optimal in all datasets and all settings but, rather, are only optimal within the assumed model. In fact, OBRC with homoscedastic arbitrary covariances (not shown in the figures) performs as well as, or significantly better than, LDA, suggesting that covariances in this problem are approximately homoscedastic. From the Q-Q plots, *Z* deviates from the reference standard normal CDF, with a clear shift in the mean and sometimes variance. For instance, under the LDA classification with *D*=2 and *n*=70 (corresponding to Fig. 9 c), the mean of *Z* is 0.76 and the standard deviation is 1.08, and under the OBRC classification with *D*=5 and *n*=70 (corresponding to Fig. 10 i), the mean of *Z* is −1.08 and the standard deviation is 1.49.

**Fig. 11:**
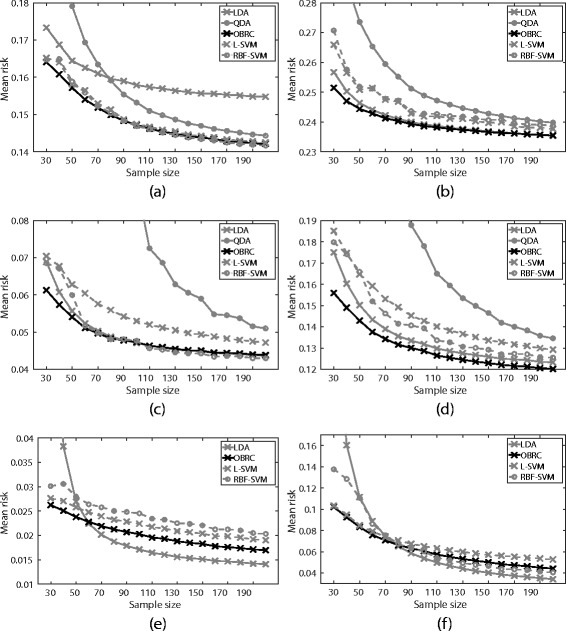
True risk mean for the TCGA dataset and five classification rules (LDA, QDA, OBRC, L-SVM, and RBF-SVM). **a** FS-1, *D*=2; **b** FS-2, *D*=2; **c** FS-1, *D*=5; **d** FS-2, *D*=5; **e** FS-1, *D*=20; **f** FS-2, *D*=20

In Fig. 11, we present the mean of the true risk with respect to random samples from the TCGA dataset, as a function of sample size, for different feature selection methods and selected feature set sizes. Due to covariance estimation problems, QDA cannot be trained for *D*=20 in this range of sample sizes. OBRC with calibrated priors consistently outperforms under small samples and performs robustly under large samples. These results depend on the particular features selected and note LDA may have an advantage under FS-2, which minimizes the apparent error of LDA classifiers.

In real applications, data rarely satisfy modeling assumptions, for instance, Gaussianity, and there may be a concern that performance will suffer. Firstly, keep in mind the need to validate assumptions in the Bayesian model. For example, Gaussianity tests and homoscedasticity tests may be used to validate these underlying assumptions. Our real-data simulations demonstrate a few examples of how Gaussianity tests may be used in conjunction with Bayesian methods. Secondly, previous works have shown that Bayesian methods are relatively robust to deviations from a Gaussianity assumption [10, 14]. This is observed, for instance, in Figs. 9 and 10. Thirdly, inference from non-informative priors may serve as a reference. The OBRC under non-informative priors and an arbitrary homoscedastic covariance model behaves similarly to LDA and under an arbitrary independent covariance model behaves similarly to QDA [13, 14]. Thus, the OBRC can be seen as unifying and optimizing these classifiers. This applies in Fig. 11, where OBRC with an appropriate covariance model and non-informative prior performs indistinguishably from LDA. The conditional MSE is also an immensely useful tool to quantify the accuracy of a risk estimator. For instance, one may employ the MSE for censored sampling by collecting batches of sample points until the sample-conditioned MSE reaches an acceptable level, and either an acceptable risk has been achieved or it has been determined that an acceptable risk cannot be achieved. Lastly, although we provide analytic solutions under discrete and Gaussian models, the basic theory for this work does not require these assumptions. For instance, recent work in [30] develops a Bayesian Poisson model for RNA-Seq data, where Bayesian error estimators and optimal Bayesian classifiers are obtained using Markov chain Monte Carlo (MCMC) techniques.

## Conclusion

We have extended optimal Bayesian classification theory to multiple classes and arbitrary loss functions, giving rise to Bayesian risk estimators, the sample-conditioned MSE for arbitrary risk estimators, and optimal Bayesian risk classifiers. We have developed a new interpretation of the conditional MSE based on effective joint densities, which is useful in developing analytic forms and approximations for the conditional MSE. We also provide new analytic solutions for the conditional MSE under homoscedastic covariance models. Simulations based on several synthetic Gaussian models and two real microarray datasets also demonstrate good performance relative to existing methods.

## Appendix 1: Discrete models

Consider a discrete sample space, $\mathcal {X} = \{1, 2, \ldots, b\}$. Let ${p_{x}^{y}}$ be the probability that a point from class *y* is observed in bin $x\in \mathcal {X}$, and let ${U_{x}^{y}}$ be the number of sample points observed from class *y* in bin *x*. Note $n_{y}=\sum _{x=1}^{b}{U_{x}^{y}}$. The discrete Bayesian model defines $\Theta _{y}=[\!{P_{1}^{y}},\ldots, {P_{b}^{y}}]$, with parameter space $\mathcal {T}_{y} = \Delta ^{b-1}$. For each *y*, we define Dirichlet priors on *Θ*_*y*_ with hyperparameters $\mathbf {\alpha }^{y} = \{{\alpha _{1}^{y}}, \ldots, {\alpha _{b}^{y}}\}$:

$$\pi (\theta_{y}) \propto \prod_{x=1}^{b}({p_{x}^{y}})^{{\alpha_{x}^{y}}-1}. $$

Assume that *Θ*_*y*_ are mutually independent. Uniform priors are achieved when ${\alpha _{x}^{y}}=1$ for all *x* and *y*. Given data, the posteriors are again Dirichlet with updated hyperparameters, $\alpha _{x}^{y\ast } = {\alpha _{x}^{y}} + {U_{x}^{y}}$ for all *x* and *y*. For proper posteriors, $\alpha _{x}^{y\ast }$ must all be positive for all *x* and *y*. The effective density is thus given by:

(31) $$\begin{array}{*{20}l} f(x \, | \, y, S) &= {\text{E}[P_{x}^{y}} \, | \, S] = \frac{\alpha_{x}^{y\ast}}{\alpha_{+}^{y\ast}},  \end{array} $$

where $\alpha _{+}^{y\ast } = \sum _{x = 1}^{b} \alpha _{x}^{y\ast }$. Thus, we have

$$\begin{array}{*{20}l} \widehat{\varepsilon }^{i,y}\left(\psi, S\right) &= \sum_{x=1}^{b} \frac{\alpha_{x}^{y\ast}}{\alpha_{+}^{y\ast}} I_{\psi(x)=i}. \end{array} $$

The effective joint density, *f*(*x*,*w* | *y*,*z*,*S*), for *y*=*z*, can be found from properties of Dirichlet distributions. We have for any *y*∈{0,…,*M*−1} and $x, w \in \mathcal {X}$,

$$\begin{array}{*{20}l} f(x, w \, | \,y, y, S) &= \text{E}\left[ {P_{x}^{y}} {P_{w}^{y}} \,| \, S \right] = \frac{\alpha_{x}^{y\ast} (\alpha_{w}^{y\ast} + \delta_{xw})}{\alpha_{+}^{y\ast}\left(\alpha_{+}^{y\ast} + 1\right)}, \end{array} $$

where *δ*_*xw*_ equals 1 if *x*=*w* and 0 otherwise. From (28),

(32) $$\begin{array}{*{20}l} &\text{E}\left[ \varepsilon^{i,y}(\Theta_{y}) \varepsilon^{j,y}(\Theta_{y}) \, | \, S\right]  \\ &\qquad = \sum_{x = 1}^{b} \sum_{w = 1}^{b} \frac{\alpha_{x}^{y\ast} \left(\alpha_{w}^{y\ast} + \delta_{xw} \right)} {\alpha_{+}^{y\ast} (\alpha_{+}^{y\ast} + 1)} I_{\psi(x) = i} I_{\psi(w) = j}  \\ &\qquad = \frac{\widehat{\varepsilon}^{i,y}(\psi, S) \left(\alpha_{+}^{y\ast} \widehat{\varepsilon}^{j,y}(\psi, S) + \delta_{ij}\right) } {\alpha_{+}^{y\ast} + 1}. \end{array} $$

When *y*≠*z*, $\text {E}\left [ \varepsilon _{n}^{i,y}(\Theta _{y}) \varepsilon _{n}^{j,z}(\Theta _{y}) \, | \, S\right ]$ may be found from (25).

## Appendix 2: Gaussian models

Suppose $\mathcal {X}$ is a *D* dimensional space in which each point is represented by a column vector and each class-*y* conditional distribution is Gaussian with mean vector **μ**_*y*_ and covariance matrix **Σ**_*y*_. We will consider independent covariance models, where the **Σ**_*y*_ are mutually independent prior to observing the data, and homoscedastic covariance models, where **Σ**_*y*_ are identical for all *y* [13]. We will also consider three structures for the covariance: known, scaled identity, and arbitrary. Throughout, we use **μ**_*y*_ and **Σ**_*y*_ to denote both random quantities and their realizations, and we use **Σ**_*y*_≻0 to denote a valid covariance matrix, i.e., a symmetric, positive definite matrix. Throughout, we will find analytic forms for the BRE and conditional MSE under binary linear classifiers, *ψ*, of the form

(33) $$ \psi(\mathbf{x}) = \left\{ \begin{array}{ll}0~~\text{if}~ g(\mathbf{x}) \leq 0,\\ 1 ~~\text{otherwise}{,} \end{array} \right.   $$

where *g*(**x**)=**a** ^*T*^ **x**+*b* for some vector **a** and scalar *b*, and a superscript *T* denotes matrix transpose.

### Known covariance

Assume that **Σ**_*y*_≻0 is known so that *Θ*_*y*_=**μ**_*y*_ with parameter space $\mathcal {T}_{y} = \mathbb {R}^{D}$. We assume the **μ**_*y*_s are mutually independent and use the following prior:

(34) $$ \pi (\mathbf{\mu}_{y}) \propto \left|\mathbf{\Sigma}_{y}\right|^{-\frac{1}{2}} \exp \left(- \frac{\nu_{y}}{2}\left(\mathbf{\mu}_{y} -\mathbf{m}_{y}\right)^{T} \mathbf{\Sigma}_{y}^{-1}\left(\mathbf{\mu}_{y} -\mathbf{m}_{y}\right) \right),   $$

with hyperparameters $\nu _{y} \in \mathbb {R}$ and $\mathbf {m}_{y} \in \mathbb {R}^{D}$, where |·| denotes a determinant. When *ν*_*y*_>0, this is a Gaussian distribution with mean **m**_*y*_ and covariance **Σ**_*y*_/*ν*_*y*_. Under this model, the posterior is of the same form as the prior, with updated hyperparameters

(35) $$\begin{array}{*{20}l} \nu_{y}^{\ast} &= \nu_{y} + n_{y},  \\ \mathbf{m}_{y}^{\ast} &= \mathbf{m}_{y} + n_{y} \frac{\widehat{\mathbf{\mu} }_{y} - \mathbf{m}_{y}}{\nu_{y}+n_{y}},  \end{array} $$

where $\widehat {\mathbf {\mu } }_{y}$ is the usual sample mean of training points in class *y*. We require $\nu _{y}^{\ast } > 0$ for a proper posterior.

The effective density was shown in [13] to be the following Gaussian distribution:

(36) $$ f(\mathbf{x} \, | \, y, S) \sim \mathcal{N} \left(\mathbf{m}^{\ast}_{y}, \frac{\nu^{\ast}_{y} + 1}{\nu^{\ast}_{y}} \mathbf{\Sigma}_{y}\right).   $$

To find the BRE for a linear classifier, let *P*=(−1)^*i*^ *g*(**X**). From the effective density,

(37) $$ f(p \, | \, y, S) \sim \mathcal{N} \left((-1)^{i} g(\mathbf{m}^{\ast}_{y}), \frac{\nu^{\ast}_{y} + 1}{\nu^{\ast}_{y}} \mathbf{a}^{T} \mathbf{\Sigma}_{y} \mathbf{a}\right).  $$

Thus,

(38) $$\begin{array}{*{20}l} \widehat{\varepsilon}^{i,y}(\psi, S) &= \text{P}((-1)^{i} g(\mathbf{X}) \leq 0 \, | \,y, S)  \\ &= \text{P}(P \leq 0 \, | \, y, S)  \\ &= \Phi\left(- \frac{(-1)^{i} g(\mathbf{m}^{\ast}_{y})}{\sqrt{\mathbf{a}^{T} \mathbf{\Sigma}_{y} \mathbf{a}}} \sqrt{\frac{\nu^{\ast}_{y}}{\nu^{\ast}_{y} + 1}} \right),  \end{array} $$

where *Φ*(*x*) is the standard normal CDF. This result was also found in [10].

To find the MSE under linear classification, note *f*(**w** | **x**,*y*,*z*,*S*) is of the same form as *f*(**x** | *y*,*S*) with posterior hyperparameters updated with {**x**,*y*} as a new sample point. Hence, for *y*=*z*,

(39) $$ f(\mathbf{w} \, | \, \mathbf{x}, y, y, S) \sim \mathcal{N} \left(\mathbf{m}^{\ast}_{y} + \frac{\mathbf{x} - \mathbf{m}^{\ast}_{y}}{\nu^{\ast}_{y} + 1}, \frac{\nu^{\ast}_{y} + 2}{\nu^{\ast}_{y} + 1} \mathbf{\Sigma}_{y} \right),   $$

and the effective joint density is thus given by

(40) $$ f(\mathbf{x}, \mathbf{w} \, | \, y, y, S) \sim \mathcal{N} \left(\left[ \begin{array}{ll} \mathbf{m}^{\ast}_{y} \\ \mathbf{m}^{\ast}_{y} \\ \end{array} \right],\left[ \begin{array}{ll} \frac{\nu^{\ast}_{y} + 1}{\nu^{\ast}_{y}} \mathbf{\Sigma}_{y} & \frac{1}{\nu^{\ast}_{y}} \mathbf{\Sigma}_{y} \\ \frac{1}{\nu^{\ast}_{y}} \mathbf{\Sigma}_{y} & \frac{\nu^{\ast}_{y} + 1}{\nu^{\ast}_{y}} \mathbf{\Sigma}_{y} \\ \end{array} \right] \right).   $$

Now let *Q*=(−1)^*j*^ *g*(**W**). Since **X** and **W** are governed by the effective joint density in (40):

$$\begin{array}{*{20}l} &f(p, q\,| \, y, y, S) \sim  \\ &\mathcal{N} \left(\left[ \begin{array}{ll} (-1)^{i} g(\mathbf{m}^{\ast}_{y}) \\ (-1)^{j} g(\mathbf{m}^{\ast}_{y}) \\ \end{array} \right], \left[ \begin{array}{ll} \frac{\nu^{\ast}_{y} + 1}{\nu^{\ast}_{y}} \mathbf{a}^{T} \mathbf{\Sigma}_{y} \mathbf{a} & \frac{(-1)^{i+j}}{\nu^{\ast}_{y}} \mathbf{a}^{T} \mathbf{\Sigma}_{y} \mathbf{a} \\ \frac{(-1)^{i+j}}{\nu^{\ast}_{y}} \mathbf{a}^{T} \mathbf{\Sigma}_{y} \mathbf{a} & \frac{\nu^{\ast}_{y} + 1}{\nu^{\ast}_{y}} \mathbf{a}^{T} \mathbf{\Sigma}_{y} \mathbf{a} \\ \end{array} \right] \right).  \end{array} $$

Hence, from (29), we have

$${} {\fontsize{8.1}{12}{\begin{aligned} \text{E} \left[ \varepsilon^{i,y}(\psi, \Theta_{y}) \varepsilon^{j,y}(\psi, \Theta_{y}) \, | \, S \right] &= \text{P}(P \leq 0 \cap Q \leq 0 \,| \, y, S)\\ &= \Phi\left(- \frac{(-1)^{i} g(\mathbf{m}^{\ast}_{y})}{\sqrt{\mathbf{a}^{T} \mathbf{\Sigma}_{y} \mathbf{a}}} \sqrt{\frac{\nu^{\ast}_{y}}{\nu^{\ast}_{y} + 1}}, \right.\\ &\quad\left.- \frac{(-1)^{j} g(\mathbf{m}^{\ast}_{y})}{\sqrt{\mathbf{a}^{T} \mathbf{\Sigma}_{y} \mathbf{a}}} \sqrt{\frac{\nu^{\ast}_{y}}{\nu^{\ast}_{y} + 1}}, \frac{(-1)^{i+j}}{\nu_{y}^{\ast}+1}\right), \end{aligned}}} $$

where *Φ*(*x*,*y*,*ρ*) is the joint CDF of two standard normal random variables with correlation *ρ*. When *y*≠*z*, E[*ε* ^*i*,*y*^(*ψ*,*Θ*_*y*_)*ε* ^*j*,*z*^(*ψ*,*Θ*_*z*_) | *S*] is found from (25).

### Homoscedastic arbitrary covariance

Assume *Θ*_*y*_=[**μ**_*y*_,**Σ**], where the parameter space of **μ**_*y*_ is $\mathbb {R}^{D}$ and the parameter space of **Σ** consists of all symmetric positive definite matrices. Further, assume a conjugate prior in which the **μ**_*y*_s are mutually independent given *Σ* so that

(41) $$ \pi (\theta)=\left(\prod_{y = 0}^{M-1} \pi (\mathbf{\mu}_{y} \, | \, \mathbf{\Sigma})\right)\pi (\mathbf{\Sigma}),  $$

where *π*(**μ**_*y*_ | **Σ**) is as in (34) with hyperparameters $\nu _{y} \in \mathbb {R}$ and $\mathbf {m}_{y} \in \mathbb {R}^{D}$, and

(42) $$\begin{array}{*{20}l} \pi (\mathbf{\Sigma})& \propto \left|\mathbf{\Sigma}\right|^{-\frac{\kappa +D+1}{2}} \exp \left(- \frac{1}{2} \text{trace} \left(\mathbf{S} \mathbf{\Sigma}^{-1}\right) \right),  \end{array} $$

with hyperparameters $\kappa \in \mathbb {R}$ and **S**, a symmetric *D*×*D* matrix. If *ν*_*y*_>0, then *π*(**μ**_*y*_ | **Σ**) is Gaussian with mean **m**_*y*_ and covariance **Σ**/*ν*_*y*_. If *κ*>*D*−1 and **S**≻0, then *π*(**Σ**) is an inverse-Wishart distribution with hyperparameters *κ* and **S**. If in addition *κ*>*D*+1, the mean of *Σ* exists and is given by E[**Σ**]=**S**/(*κ*−*D*−1); thus, **S** determines the shape of the expected covariance. The posterior is of the same form as the prior with the same updated hyperparameters given by (35) and

(43) $$\begin{array}{*{20}l} \kappa^{\ast} &=\kappa +n,  \\ \mathbf{S}^{\ast} &= \mathbf{S} + \sum_{y = 0}^{M-1} (n_{y}-1)\widehat{\mathbf{\Sigma} }_{y} + \textstyle\frac{\nu_{y}n_{y}}{\nu_{y}+n_{y}}(\widehat{\mathbf{\mu} }_{y}-\mathbf{m}_{y})(\widehat{\mathbf{\mu} }_{y}-\mathbf{m}_{y})^{T},  \end{array} $$

where $\widehat {\mathbf {\Sigma } }_{y}$ is the usual sample covariance of training points in class *y* ($\widehat {\mathbf {\Sigma }}_{y} = 0$ if *n*_*y*_≤1). The posteriors are proper if $\nu _{y}^{\ast } >0$, *κ* ^∗^>*D*−1 and **S** ^∗^≻0.

The effective density for class *y* is multivariate student *t* with *k*=*κ* ^∗^−*D*+1 degrees of freedom, location vector $\mathbf {m}_{y}^{\ast }$, and scale matrix $\frac {\nu _{y}^{\ast }+1}{k \nu _{y}^{\ast }} \mathbf {S}^{\ast }$ [13]. In other words,

(44) $$ f(\mathbf{x} \, | \, y, S) \sim t \left(k, \mathbf{m}_{y}^{\ast}, \frac{\nu_{y}^{\ast}+1}{k \nu_{y}^{\ast}} \mathbf{S}^{\ast} \right).   $$

To find the BRE under a binary linear classifier of the form (33), let *P*=(−1)^*i*^ *g*(**X**). Since *P* is an affine transformation of a multivariate student *t* random variable, it has a non-standardized student *t* distribution [31]:

(45) $$ f(p \, | \, y, S) \sim t \left(k, m_{iy}, \frac{\nu_{y}^{\ast}+1}{k \nu_{y}^{\ast}} \gamma^{2} \right),   $$

where $m_{\textit {iy}} = (-1)^{i} g(\mathbf {m}_{y}^{\ast })$ and *γ* ^2^=**a** ^*T*^ **S** ^∗^ **a**. The CDF of a non-standardized student *t* distribution with *d* degrees of freedom, location parameter *m*, and scale parameter *s* ^2^ is well known, and at zero, it is given by [32],

$$\frac{1}{2} - \frac{\text{sgn}(m)}{2} I\left(\frac{m^{2}}{m^{2} + d s^{2}}; \frac{1}{2}, \frac{d}{2}\right), $$

where *I*(*x*;*a*,*b*) is an incomplete regularized beta function. Hence,

(46) $$\begin{array}{*{20}l} \widehat{\varepsilon}^{i,y}(\psi, S) &= \frac{1}{2} - \frac{\text{sgn}(m_{iy})}{2} I\left(\frac{m_{iy}^{2}}{m_{iy}^{2} + \frac{\nu_{y}^{\ast}+1}{\nu_{y}^{\ast}} \gamma^{2}}; \frac{1}{2}, \frac{k}{2}\right).  \end{array} $$

This result was also found in [10].

The effective conditional density for *y*=*z* is solved by updating all of the hyperparameters associated with class *y* with the new sample point, {**x**,*y*}, resulting in:

(47) $$\begin{array}{*{20}l} &f(\mathbf{w} \, | \, \mathbf{x}, y, y, S) \sim t \left(k+1, \mathbf{m}_{y}^{\ast} + \frac{\mathbf{x} - \mathbf{m}_{y}^{\ast }}{\nu_{y}^{\ast}+1}, \right.  \\ & \qquad\qquad\qquad \left. \frac{\nu^{\ast }_{y}+2}{(k+1)(\nu^{\ast }_{y} + 1)}\left(\mathbf{S}^{\ast} + \mathbf{S}_{y}(\mathbf{x})\right) \right),  \end{array} $$

where

(48) $$ \mathbf{S}_{y}(\mathbf{x}) = \frac{\nu_{y}^{\ast}}{\nu_{y}^{\ast}+1}(\mathbf{x}-\mathbf{m}_{y}^{\ast})(\mathbf{x}-\mathbf{m}_{y}^{\ast})^{T}.   $$

For *y*≠*z*, *f*(**w** | **x**,*y*,*z*,*S*) is of the same form as the effective density with only hyperparameters associated with the covariance, *κ* ^∗^ and **S** ^∗^, updated:

(49) $$\begin{array}{*{20}l} &f(\mathbf{w} \,| \, \mathbf{x}, y, z, S) \sim t \left(k+1, \mathbf{m}_{z}^{\ast }, \right.  \\ & \quad\qquad\qquad\qquad \left. \frac{\nu^{\ast }_{z}+1}{(k + 1)\nu^{\ast }_{z}} \left(\mathbf{S}^{\ast} + \mathbf{S}_{y}(\mathbf{x})\right) \right).  \end{array} $$

To find the conditional MSE of the BRE, let *Q*=(−1)^*j*^ *g*(**W**). For *y*=*z*,

(50) $$\begin{array}{*{20}l} &f(q \,| \, \mathbf{x}, y, y, S) \sim t \left(k+1, m_{iy} + \frac{p - m_{iy}}{\nu_{y}^{\ast} + 1}, \right.  \\ &\left. \frac{\nu_{y}^{\ast}+2}{(k+1)(\nu_{y}^{\ast}+1)} \left(\gamma^{2} + \frac{\nu_{y}^{\ast}}{\nu_{y}^{\ast}+1} \left(p - m_{iy} \right)^{2} \right) \right),  \end{array} $$

where we have used the fact that $(-1)^{i} \mathbf {a}^{T} (\mathbf {x} - \mathbf {m}_{y}^{\ast }) = p - m_{y}$. When *y*≠*z*,

$$\begin{array}{*{20}l} f(q \, | \, \mathbf{x}, y, z, S) &\sim t \left(k+1, m_{jz}, \right. \\ & \left. \frac{\nu_{z}^{\ast}+1}{(k+1)\nu_{z}^{\ast}} \left(\gamma^{2} + \frac{\nu_{y}^{\ast}}{\nu_{y}^{\ast}+1} \left(p - m_{iy} \right)^{2} \right) \right). \end{array} $$

Since dependency on **X** has been reduced to dependency on only *P* in both of the above distributions, we may write *f*(*q* | **x**,*y*,*z*,*S*)=*f*(*q* | *p*,*y*,*z*,*S*) for all *y* and *z*. Lemma 1 in Appendix Appendix 3: Effective joint density lemma produces an effective joint density given an effective density and an effective conditional density of a specified form. The distributions *f*(*p* | *y*,*S*) and *f*(*q* | *p*,*y*,*y*,*S*) are precisely in the form required by this lemma with *D*=1. Hence, [*P*,*Q*]^*T*^ follows a bivariate student *t* distribution when *y*=*z*,

(51) $$ f(p, q \, | \, y, y, S) \sim t\left(k,\left[ \begin{array}{ll} m_{iy} \\ m_{iy} \\ \end{array} \right], \frac{\gamma^{2}}{k} \left[ \begin{array}{ll} \frac{\nu_{y}^{\ast} + 1}{\nu_{y}^{\ast}} & \frac{(-1)^{i+j}}{\nu_{y}^{\ast}} \\ \frac{(-1)^{i+j}}{\nu_{y}^{\ast}} & \frac{\nu_{y}^{\ast} + 1}{\nu_{y}^{\ast}} \\ \end{array} \right] \right),   $$

and when *y*≠*z*,

$$f(p, q \,| \, y, z, S) \sim t\left(k, \left[ \begin{array}{ll} m_{iy} \\ m_{jz} \\ \end{array} \right], \frac{\gamma^{2}}{k} \left[ \begin{array}{cc} \frac{\nu_{y}^{\ast} + 1}{\nu_{y}^{\ast}} & 0 \\ 0 & \frac{\nu_{z}^{\ast} + 1}{\nu_{z}^{\ast}}\\ \end{array} \right] \right). $$

Thus, E[*ε* ^*i*,*y*^(*Θ*_*y*_)*ε* ^*j*,*y*^(*Θ*_*y*_) | *S*] can be found from (29). In particular, when *y*=*z*,

(52) $$\begin{array}{@{}rcl@{}} \text{E} \left[ \varepsilon^{i,y}(\Theta_{y}) \varepsilon^{j,y}(\Theta_{y}) \, | \, S \right] &= \text{P}(P \leq 0 \cap Q \leq 0 \,| \, y, y, S)  \\ &= \mathbf{T}\left(- \frac{m_{iy}}{\gamma} \sqrt{\frac{k \nu_{y}^{\ast}}{\nu_{y}^{\ast} + 1}}, - \frac{m_{jy}}{\gamma}\right.\\ &\quad\left.\sqrt{\frac{k \nu_{y}^{\ast}}{\nu_{y}^{\ast} + 1}}, \frac{(-1)^{i+j}}{\nu_{y}^{\ast}+1}, k \right),  \end{array} $$

and when *y*≠*z*,

(53) $$\begin{array}{@{}rcl@{}} \text{E} \left[ \varepsilon^{i,y}(\Theta_{y}) \varepsilon^{j,z}(\Theta_{z}) \, | \, S \right] &=\text{P} \text{}(P \leq 0 \cap Q \leq 0 \, | \, y, z, S) \\ &= \mathbf{T}\left(- \frac{m_{iy}}{\gamma} \sqrt{\frac{k \nu_{y}^{\ast}}{\nu_{y}^{\ast} + 1}}, - \frac{m_{jz}}{\gamma}\right. \\ &\quad\left.\sqrt{\frac{k \nu_{z}^{\ast}}{\nu_{z}^{\ast} + 1}}, 0, k \right), \end{array} $$

where **T**(*x*,*y*,*ρ*,*d*) is the joint CDF of two standard multivariate student *t* random variables with correlation *ρ* and *d* degrees of freedom.

### Independent arbitrary covariance

Assume *Θ*=[ **μ**_*y*_,*Σ*_*y*_], where the parameter space of **μ**_*y*_ is $\mathbb {R}^{D}$ and the parameter space of **Σ**_*y*_ consists of all symmetric positive definite matrices. The independent arbitrary covariance model assumes a conjugate prior with independent *Θ*_*y*_s and

(54) $$ \pi (\theta_{y})=\pi (\mathbf{\mu}_{y} \, | \, \mathbf{\Sigma}_{y})\pi (\mathbf{\Sigma}_{y}),  $$

where *π*(**μ**_*y*_ | **Σ**_*y*_) is of the same form as in (34) with hyperparameters $\nu _{y} \in \mathbb {R}$ and $\mathbf {m}_{y} \in \mathbb {R}^{D}$, and *π*(**Σ**_*y*_) is of the same form as in (42) with hyperparameters $\kappa _{y} \in \mathbb {R}$ and **S**_*y*_, a symmetric *D*×*D* matrix. The posterior is of the same form as the prior with updated hyperparameters given by (35) and

(55) $$\begin{array}{*{20}l} \kappa_{y}^{\ast} &=\kappa_{y}+n_{y},  \\ \mathbf{S}_{y}^{\ast} &= \mathbf{S}_{y}+(n_{y}-1)\widehat{\mathbf{\Sigma} }_{y} + \textstyle \frac{\nu_{y}n_{y}}{\nu_{y}+n_{y}}(\widehat{\mathbf{\mu} }_{y}-\mathbf{m}_{y})(\widehat{\mathbf{\mu} }_{y}-\mathbf{m}_{y})^{T}.  \end{array} $$

The posteriors are proper if $\nu _{y}^{\ast } >0$, $\kappa _{y}^{\ast } >D-1$ and $\mathbf {S}_{y}^{\ast } \succ 0$.

The effective density for class *y* is multivariate student *t* as in (44) with $k_{y} = \kappa _{y}^{\ast }-D+1$ and $\mathbf {S}_{y}^{\ast }$ in place of *k* and **S** ^∗^, respectively [13]. Further, (45) also holds with $m_{\textit {iy}} = (-1)^{i} g(\mathbf {m}_{y}^{\ast })$ and with *k*_*y*_ and ${\gamma _{y}^{2}} = \mathbf {a}^{T} \mathbf {S}_{y}^{\ast } \mathbf {a}$ in place of *k* and *γ* ^2^, respectively. Under binary linear classification, $\widehat {\varepsilon }^{i,y}(\psi, S)$ is given by (46) with *k*_*y*_ and ${\gamma _{y}^{2}}$ in place of *k* and *γ* ^2^. The same result was found in [10]. E[*ε* ^*i*,*y*^(*Θ*_*y*_)*ε* ^*j*,*y*^(*Θ*_*y*_) | *S*] is solved similarly to before, resulting in (47), (50), (51), and ultimately (52), with *k*_*y*_, $\mathbf {S}_{y}^{\ast }$ and ${\gamma _{y}^{2}}$ in place of *k*, **S** ^∗^, and *γ* ^2^, respectively. E[*ε* ^*i*,*y*^(*Θ*_*y*_)*ε* ^*j*,*z*^(*Θ*_*z*_) | *S*] for *y*≠*z* is found from (25).

### Homoscedastic scaled identity covariance

In the homoscedastic scaled identity covariance model, **Σ**_*y*_ is assumed to have a scaled identity structure, i.e., *Θ*_*y*_=[**μ**_*y*_,*σ* ^2^] where **Σ**_*y*_=*σ* ^2^ **I**_*D*_ and **I**_*D*_ is a *D*×*D* identity matrix. The parameter space of **μ**_*y*_ is $\mathbb {R}^{D}$ for all *y* and of *σ* ^2^ is (0,*∞*). We also assume the **μ**_*y*_s are mutually independent given *σ* ^2^:

(56) $$ \pi (\theta)=\left(\prod_{y=0}^{M-1} \pi (\mathbf{\mu}_{y} \, | \, \sigma^{2})\right) \pi (\sigma^{2}),  $$

where *π*(**μ**_*y*_ | *σ* ^2^) is of the same form as (34) with hyperparameters *ν*_*y*_ and **m**_*y*_, and

(57) $$\begin{array}{*{20}l} \pi (\sigma^{2})& \propto \left\vert \sigma^{2} \right\vert^{-\frac{(\kappa +D+1)D}{2}} \exp \left(- \frac{\text{trace} (\mathbf{S})}{2 \sigma^{2}} \right),  \end{array} $$

with hyperparameters $\kappa \in \mathbb {R}$ and **S**, a symmetric *D*×*D* real matrix. When *ν*_*y*_>0, *π*(**μ**_*y*_ | *σ* ^2^) is a univariate Gaussian distribution with mean **m**_*y*_ and covariance **Σ**_*y*_/*ν*_*y*_, and when (*κ*+*D*+1)*D*>2 and **S**≻0, *π*(*σ* ^2^) is a univariate inverse-Wishart distribution. If in addition (*κ*+*D*+1)*D*>4, then $\text {E}[\sigma ^{2}] = \frac {\text {trace} (\mathbf {S})}{(\kappa +D+1)D - 4}$. The form of (57) has been designed so that the posterior is of the same form as the prior with the same hyperparameter update equations given in the arbitrary covariance models, (35) and (43). We require $\nu _{y}^{\ast } > 0$, (*κ* ^∗^+*D*+1)*D*>2, and **S** ^∗^≻0 for a proper posterior.

The effective density for class *y* is multivariate student *t* with *k*=(*κ* ^∗^+*D*+1)*D*−2 degrees of freedom [13]:

(58) $$ f(\mathbf{x} \, | \, y, S) \sim t \left(k, \mathbf{m}_{y}^{\ast}, \frac{\nu_{y}^{\ast}+1}{k \nu_{y}^{\ast}} \text{trace} (\mathbf{S}^{\ast}) \mathbf{I}_{D}\right).   $$

Let *P*=(−1)^*i*^ *g*(**X**). Since *P* is an affine transformation of a multivariate student *t* random variable, again it has the same form as in (45) with *k*=(*κ* ^∗^+*D*+1)*D*−2, $m_{\textit {iy}} = (-1)^{i} g(\mathbf {m}_{y}^{\ast })$, and *γ* ^2^=trace(**S** ^∗^)**a** ^*T*^ **a**. Following the same steps as in the homoscedastic arbitrary covariance model, under binary linear classification, $\widehat {\varepsilon }^{i,y}(\psi, S)$ is given by (46) with the appropriate choice of *k*, *m*_*iy*_, and *γ* ^2^. This was found in [10].

The effective conditional density for *y*=*z* is solved by updating all of the hyperparameters associated with class *y* with the new sample point, {**x**,*y*}:

(59) $$\begin{array}{*{20}l} &f(\mathbf{w} \, | \, \mathbf{x}, y, y, S) \sim t \Big(k + D, \mathbf{m}_{y}^{\ast }+\frac{\mathbf{x} - \mathbf{m}_{y}^{\ast }}{\nu_{y}^{\ast}+1},  \\ &\qquad \frac{\nu_{y}^{\ast}+2}{(k+D) (\nu_{y}^{\ast}+1)} \text{trace} (\mathbf{S}^{\ast} + \mathbf{S}_{y}(\mathbf{x})) \mathbf{I}_{D} \Big),  \end{array} $$

where **S**_*y*_(**x**) is given by (48). When *y*≠*z*, the effective conditional density is found by updating only hyperparameters associated with the covariance, *κ* ^∗^ and **S** ^∗^, with the point {**x**,*y*}. Thus,

(60) $$\begin{array}{*{20}l} &f(\mathbf{w} \,| \, \mathbf{x}, y, z, S) \sim t \left(k + D, \mathbf{m}_{z}^{\ast }, \right.  \\ &\qquad\qquad\qquad \left. \frac{\nu_{z}^{\ast}+1}{(k+D) \nu_{z}^{\ast}} \text{trace} (\mathbf{S}^{\ast} + \mathbf{S}_{y}(\mathbf{x})) \mathbf{I}_{D} \right).  \end{array} $$

Lemma 1 in Appendix Appendix 3: Effective joint density lemma is used to find an effective joint density. When *y*=*z*,

(61) $$\begin{array}{*{20}l} & f(\mathbf{x}, \mathbf{w} \, | \, y, y, S)  \\ &\quad\sim t\left(k, \left[ \begin{array}{ll} \mathbf{m}_{y}^{\ast} \\ \mathbf{m}_{y}^{\ast} \\ \end{array} \right], \frac{\text{trace} (\mathbf{S}^{\ast})}{k} \left[ \begin{array}{ll} \frac{\nu_{y}^{\ast} + 1}{\nu_{y}^{\ast}} \mathbf{I}_{D} & \frac{1}{\nu_{y}^{\ast}} \mathbf{I}_{D} \\ \frac{1}{\nu_{y}^{\ast}} \mathbf{I}_{D} & \frac{\nu_{y}^{\ast} + 1}{\nu_{y}^{\ast}} \mathbf{I}_{D} \\ \end{array} \right] \right),  \end{array} $$

and when *y*≠*z*,

(62) $$\begin{array}{*{20}l} & f(\mathbf{x}, \mathbf{w} \, | \, y, z, S)  \\ &\quad\sim t\left(k, \left[ \begin{array}{ll} \mathbf{m}_{y}^{\ast} \\ \mathbf{m}_{z}^{\ast} \\ \end{array} \right], \frac{\text{trace} (\mathbf{S}^{\ast})}{k} \left[ \begin{array}{cc} \frac{\nu_{y}^{\ast} + 1}{\nu_{y}^{\ast}} \mathbf{I}_{D} & \mathbf{0}_{D} \\ \mathbf{0}_{D} & \frac{\nu_{z}^{\ast} + 1}{\nu_{z}^{\ast}} \mathbf{I}_{D} \\ \end{array} \right] \right).  \end{array} $$

E[*ε* ^*i*,*y*^(*Θ*_*y*_)*ε* ^*j*,*z*^(*Θ*_*y*_) | *S*] can be found from (29) by defining *P*=(−1)^*i*^ *g*(**X**) and *Q*=(−1)^*j*^ *g*(**W**). Following the same steps as in the homoscedastic arbitrary covariance model, one can show that E[*ε* ^*i*,*y*^(*Θ*_*y*_)*ε* ^*j*,*z*^(*Θ*_*y*_) | *S*] is equivalent to (52) when *y*=*z* and (53) when *y*≠*z*, where we plug in appropriate values for *k*, *m*_*iy*_ and *γ* ^2^.

### Independent scaled identity covariance

Now assume that **Σ**_*y*_ has a scaled identity structure, i.e., $\Theta _{y} = [\mathbf {\mu }_{y}, {\sigma _{y}^{2}}]$ where $\mathbf {\Sigma }_{y} = {\sigma _{y}^{2}} \mathbf {I}_{D}$, and that the parameter space of **μ**_*y*_ is $\mathbb {R}^{D}$ and of ${\sigma _{y}^{2}}$ is (0,*∞*) for all *y*. Also, assume the *Θ*_*y*_s are mutually independent, with

(63) $$ \pi (\theta_{y})=\pi (\mathbf{\mu}_{y} \,| \, {\sigma_{y}^{2}})\pi ({\sigma_{y}^{2}}),  $$

where $\pi (\mathbf {\mu }_{y} \, | \, {\sigma _{y}^{2}})$ is of the same form as in (34) with hyperparameters $\nu _{y} \in \mathbb {R}$ and $\mathbf {m}_{y} \in \mathbb {R}^{D}$, and $\pi ({\sigma _{y}^{2}})$ is of the same form as in (57) with hyperparameters $\kappa _{y} \in \mathbb {R}$ and **S**_*y*_, a symmetric *D*×*D* real matrix. The posterior is of the same form as the prior with the same hyperparameter update equations in (35) and (55). We require $\nu _{y}^{\ast } > 0$, $(\kappa _{y}^{\ast } +D+1)D > 2$ and $\mathbf {S}_{y}^{\ast } \succ 0$ for a proper posterior.

The effective density for class *y* is multivariate student *t*, as in (58) with $k_{y} = (\kappa _{y}^{\ast } + D + 1)D-2$ and $\mathbf {S}_{y}^{\ast }$ in place of *k* and **S** ^∗^, respectively [13]. Under binary linear classification, $\widehat {\varepsilon }^{i,y}(\psi, S)$ is given by (46) with $m_{\textit {iy}} = (-1)^{i} g(\mathbf {m}_{y}^{\ast })$ and with *k*_*y*_ and ${\gamma _{y}^{2}} = \text {trace} (\mathbf {S}_{y}^{\ast }) \mathbf {a}^{T} \mathbf {a}$ in place of *k* and *γ* ^2^. The effective joint density, *f*(**x**,**w** | *y*,*y*,*S*), is solved as before, resulting in (59) and (61) with *k*_*y*_ and $\mathbf {S}_{y}^{\ast }$ in place of *k* and **S** ^∗^, respectively. Further, E[*ε* ^*i*,*y*^(*Θ*_*y*_)*ε* ^*j*,*y*^(*Θ*_*y*_) | *S*] is solved from (51) resulting in (52), with *k*_*y*_ and ${\gamma _{y}^{2}}$ in place of *k* and *γ* ^2^, respectively. E[*ε* ^*i*,*y*^(*Θ*_*y*_)*ε* ^*j*,*z*^(*Θ*_*z*_) | *S*] for *y*≠*z* is found from (25).

## Appendix 3: Effective joint density lemma

The lemma below is used to derive the effective joint density of Gaussian models in Appendix Appendix 2: Gaussian models.

### **Lemma1**.

Suppose **X** is multivariate student *t* given by,

$$\begin{array}{*{20}l} & f(\mathbf{x}) \sim t\left(k, \mathbf{m}_{y}^{\ast}, \frac{\nu_{y}^{\ast} + 1}{k \nu_{y}^{\ast}} \gamma^{2} \mathbf{I}_{D} \right). \end{array} $$

Further, suppose **W** conditioned on **X**=**x** is multivariate student *t* given by,

$$\begin{array}{*{20}l} & f(\mathbf{w} \, | \, \mathbf{x}) \sim t\left(k + D, \mathbf{m}_{z}^{\ast} + I \frac{\mathbf{x} - \mathbf{m}_{y}^{\ast}}{\nu_{y}^{\ast} + 1},\right. \\ &\left. \quad \frac{1}{k + D}J\left(\gamma^{2} + \frac{\nu_{y}^{\ast}}{\nu_{y}^{\ast} + 1} (\mathbf{x} - \mathbf{m}_{y}^{\ast})^{T} (\mathbf{x} - \mathbf{m}_{y}^{\ast}) \right) \mathbf{I}_{D} \right), \end{array} $$

where either *I*=0 and $J = \frac {\nu _{z}^{\ast } + 1}{\nu _{z}^{\ast }}$, or *I*=1 and $J = \frac {\nu _{y}^{\ast } + 2}{\nu _{y}^{\ast } + 1}$. Then, the joint density is multivariate student *t*:

$$\begin{array}{*{20}l} & f(\mathbf{x}, \mathbf{w}) \sim t\left(k, \left[ \begin{array}{ll} \mathbf{m}_{y}^{\ast} \\ \mathbf{m}_{z}^{\ast} \\ \end{array} \right], \frac{\gamma^{2}}{k} \left[ \begin{array}{ll} \frac{\nu_{y}^{\ast} + 1}{\nu_{y}^{\ast}} \mathbf{I}_{D} & I \frac{1}{\nu_{y}^{\ast}} \mathbf{I}_{D} \\ I \frac{1}{\nu_{y}^{\ast}} \mathbf{I}_{D} & K \mathbf{I}_{D} \\ \end{array} \right] \right), \end{array} $$

where $K = \frac {\nu _{z}^{\ast } + 1}{\nu _{z}^{\ast }}$ when *I*=0 and $K = \frac {\nu _{y}^{\ast } + 1}{\nu _{y}^{\ast }}$ when *I*=1.

### *Proof*.

After some simplification, one can show

$$\begin{array}{*{20}l} f(\mathbf{x}, \mathbf{w}) =& f(\mathbf{x}) f(\mathbf{w} \, | \, \mathbf{x}) \\ &\propto\!\! \left(\!\!1 \,+\, \frac{\nu_{y}^{\ast}}{\nu_{y}^{\ast} + 1}(\mathbf{x} \!- \!\mathbf{m}_{y}^{\ast})^{T} (\gamma^{2} \mathbf{I}_{D})^{-1} (\mathbf{x} - \mathbf{m}_{y}^{\ast})\!\!\right)\!^{-\frac{k + D}{2}} \\ & \times \left|\gamma^{2} \mathbf{I}_{D} + \frac{\nu_{y}^{\ast}}{\nu_{y}^{\ast} + 1} (\mathbf{x} - \mathbf{m}_{y}^{\ast})^{T} (\mathbf{x} - \mathbf{m}_{y}^{\ast}) \mathbf{I}_{D}\right|^{\frac{k + 2D - 1}{2}} \\ & \times \left| \gamma^{2} \mathbf{I}_{D} + \frac{\nu_{y}^{\ast}}{\nu_{y}^{\ast} + 1} (\mathbf{x} - \mathbf{m}_{y}^{\ast})^{T} (\mathbf{x} - \mathbf{m}_{y}^{\ast}) \mathbf{I}_{D} \right. \\ & + \frac{1}{J} \left(\mathbf{w} - \mathbf{m}_{z}^{\ast} - I\frac{\mathbf{x} - \mathbf{m}_{y}^{\ast}}{\nu_{y}^{\ast} + 1}\right) \\ &\left. \times \left(\mathbf{w} - \mathbf{m}_{z}^{\ast} - I\frac{\mathbf{x} - \mathbf{m}_{y}^{\ast}}{\nu_{y}^{\ast} + 1}\right)^{T} \right|^{-\frac{k + 2D}{2}}. \end{array} $$

Simplifying further, we obtain

$$\begin{array}{*{20}l} f(\mathbf{x}, \mathbf{w}) &\propto \left(\gamma^{2} + \frac{\nu_{y}^{\ast}}{\nu_{y}^{\ast} + 1} (\mathbf{x} - \mathbf{m}_{y}^{\ast})^{T} (\mathbf{x} - \mathbf{m}_{y}^{\ast}) \right. \\ & \quad + \frac{1}{J} \left(\mathbf{w} - \mathbf{m}_{z}^{\ast} - I\frac{\mathbf{x} - \mathbf{m}_{y}^{\ast}}{\nu_{y}^{\ast} + 1}\right)^{T} \\ & \quad \times \left. \left(\mathbf{w} - \mathbf{m}_{z}^{\ast} - I\frac{\mathbf{x} - \mathbf{m}_{y}^{\ast}}{\nu_{y}^{\ast} + 1}\right) \right)^{-\frac{k + 2D}{2}}. \end{array} $$

If *I*=0, then it can be shown that

$$\begin{array}{*{20}l} f(\mathbf{x}, \mathbf{w}) &\propto \left(1 + \left[ \begin{array}{ll} \mathbf{x} - \mathbf{m}_{y}^{\ast} \\ \mathbf{w} - \mathbf{m}_{z}^{\ast} \\ \end{array} \right]^{T} \mathbf{\Lambda}^{-1} \left[ \begin{array}{ll} \mathbf{x} - \mathbf{m}_{y}^{\ast} \\ \mathbf{w} - \mathbf{m}_{z}^{\ast} \\ \end{array} \right] \right)^{-\frac{k + 2D}{2}}, \end{array} $$

where

$$\mathbf{\Lambda} = \left[ \begin{array}{cc} \frac{\nu_{y}^{\ast} + 1}{\nu_{y}^{\ast}} \gamma^{2} \mathbf{I}_{D} & \mathbf{0}_{D} \\ \mathbf{0}_{D} & \frac{\nu_{z}^{\ast} + 1}{\nu_{z}^{\ast}} \gamma^{2} \mathbf{I}_{D} \\ \end{array}\right].$$

Similarly, if *I*=1, it can be shown that

$$\begin{array}{*{20}l} f(\mathbf{x}, \mathbf{w}) &\propto \left(1 + \left[ \begin{array}{ll} \mathbf{x} - \mathbf{m}_{y}^{\ast} \\ \mathbf{w} - \mathbf{m}_{z}^{\ast} \\ \end{array}\right]^{T} \mathbf{\Lambda}^{-1} \left[ \begin{array}{ll} \mathbf{x} - \mathbf{m}_{y}^{\ast} \\ \mathbf{w} - \mathbf{m}_{z}^{\ast} \\ \end{array} \right] \right)^{-\frac{k + 2 D}{2}}, \end{array} $$

where

$$\mathbf{\Lambda} = \left[ \begin{array}{ll} \frac{\nu_{y}^{\ast} + 1}{\nu_{y}^{\ast}} \gamma^{2} \mathbf{I}_{D} & \frac{1}{\nu_{y}^{\ast}} \gamma^{2} \mathbf{I}_{D} \\ \frac{1}{\nu_{y}^{\ast}} \gamma^{2} \mathbf{I}_{D} & \frac{\nu_{y}^{\ast} + 1}{\nu_{y}^{\ast}} \gamma^{2} \mathbf{I}_{D} \\ \end{array} \right],$$

which completes the proof.
